# A pyroptosis expression pattern score predicts prognosis and immune microenvironment of lung squamous cell carcinoma

**DOI:** 10.3389/fgene.2022.996444

**Published:** 2022-11-10

**Authors:** Wei Chen, Min-Yu Wen, Kai-Bin Yang, Li-Tao Zheng, Xuan Li

**Affiliations:** ^1^ State Key Laboratory of Oncology in South China, Collaborative Innovation Center for Cancer Medicine, Sun Yat-sen University Cancer Center, Guangzhou, China; ^2^ Zhongshan School of Medicine, Sun Yat-sen University, Guangzhou, China; ^3^ Department of Radiation Oncology, Sun Yat-sen University Cancer Center, State Key Laboratory of Oncology in South China, Collaborative Innovation Center of Cancer Medicine, Guangzhou, China

**Keywords:** pyroptosis, lung squamous cell carcinoma, TCGA, prognosis, immune microenvironment

## Abstract

Pyroptosis has been proved to significantly influence the development of lung squamous cell carcinoma (LUSC). To better predict overall survival (OS) and provide guidance on the selection of therapy for LUSC patients, we constructed a novel prognostic biomarker based on pyroptosis-related genes. The dataset for model construction were obtained from The Cancer Genome Atlas and the validation dataset were obtained from Gene Expression Omnibus. Differential expression genes between different pyroptosis expression patterns were identified. These genes were then used to construct pyroptosis expression pattern score (PEPScore) through weighted gene co-expression network analysis, univariate and multivariate cox regression analysis. Afterward, the differences in molecule and immune characteristics and the effect of different therapies were explored between the subgroups divided by the model. The PEPScore was constructed based on six pyroptosis-related genes (*CSF2*, *FGA*, *AKAP*12, *CYP2C18*, *IRS4*, *TSLP*). Compared with the high-PEPScore subgroup, the low-PEPScore subgroup had significantly better OS, higher *TP53* and *TTN* mutation rate, higher infiltration of T follicular helper cells and CD8 T cells, and may benefit more from chemotherapeutic drugs, immunotherapy and radiotherapy. PEPScore is a prospective prognostic model to differentiate prognosis, molecular and immune microenvironmental features, as well as provide significant guidance for selecting clinical therapies.

## Introduction

As one of the most common cancers, lung cancer accounts for a large portion of death from cancer worldwide. Although the incidence declined from 2009 to 2018, approximately 350 people die of lung cancer per day in the United States. (Siegel et al., 2022). In non-small cell lung cancer (NSCLC), lung squamous cell carcinoma accounts for approximately 25%–30% of cases. The treatment for patients with LUSC is usually considered difficult due to numerous disease features and comorbidities such as chronic obstructive pulmonary disease. (Papi et al., 2004). LUSC is not sensitive to many target therapies for the alterations approved for targeted treatments are rare. Additionally, its sensitivity to chemotherapy and radiotherapy is unsatisfactory. Thus, the options for the treatment for the LUSC are limited, especially in advanced LUSC. Given such difficulties, establishing a reliable and accurate prognostic maker which could assist in developing medical plans for LUSC is urgently needed.

Pyroptosis, initially discovered in the mononuclear macrophage, is a type of lytic inflammatory cell death initiated by the inflammasome. Gasdermins (GSDMs), pre-forming effector proteins, are the crucial mediators of pyroptosis. As the cytoplasm perceives invasive infections or danger signals, the GSDMs will be activated. Activated GSDMs are then inserted into cytomembranes and form large pores on the cytomembranes, disrupting the cell osmotic potential and inducing rapid cell death. Pyroptosis is associated with various pathophysiological effects in humans, and it has been reported to be related to hair loss, asthma and hearing impairment. (Shi et al., 2017; Liu et al., 2021). There is increasing evidence suggesting that pyroptosis could inhibit or promote tumorigenesis. For example, the expression of the GSDMD could suppress gastric cancer cell proliferation, while low expression of the GSDMD shows a suppressive effect on NSCLC cell proliferation. (Gao et al., 2018). GSDMA and GSDME are epigenetically inhibited by methylation in most human cancer cells. (Moussette et al., 2017). Nevertheless, the correlation between the pyroptosis state and the prognosis of the LUSC remains unclear.

Considering existing studies, pyroptosis is significantly influence the development of the LUSC. (Zhang et al., 2019; Liu et al., 2021). In this study, the hub differentially expressed genes (DEGs) significantly associated with pyroptosis expression pattern and OS of LUSC patients were identified by weighted gene co-expression network analysis (WGCNA) and univariate Cox regression analysis on a genome-wide scale. We constructed a novel prognostic maker, pyroptosis expression pattern score (PEPScore), for investigating the prognosis value of these genes. Then the molecular and immune profile of the PEPScore was explored. We found that the tumor environment was significantly affected by pyroptosis, and we also confirmed that the PEPScore is a promising prognostic marker and has an important guiding significance for the selection of the chemotherapy, radiotherapy and immunotherapy.

## Methods

### Patients and datasets

The mRNAs-seq data, gene mutation information and the relevant clinical data of 551 LUSC, comprising 502 cancer samples and 49 para-cancer samples, were acquired from the TCGA database (Supplementary Table S1) (https://portal.gdc.cancer.gov/repository). For the external validation cohort, Gene Expression Omnibus (GEO) was used to collect the mRNAs-seq data and related clinical data. (ID: GSE30219, GSE73403, https://www.ncbi.nlm.nih.gov/geo/).

### Identification of different pyroptosis states in LUSC and their association with survival

A total of 51 pyroptosis-related genes were gathered from the previous articles (Jiang et al., 2021; Li et al., 2021) and Molecular Signatures Database (Subramanian et al., 2005; Liberzon et al., 2015) (MSigDb, version: 7.4 http://www.gseamsigdb.org/gsea/msigdb/cards/REACTOME_PYROPTOSIS.html). We investigated the expression differences of pyroptosis-related genes between 502 tumors and 49 normal samples by utilizing the R package of “limma” with a *p*-value of 0.05. The Pearson correlation between these pyroptosis-related genes was calculated in tumor samples utilizing the “corrplot” package.

The relationship between the pyroptosis-related genes and the essential cancer pathway activity was accessed through the GSCALite website. RPPA data form TCPA database was used to calculate score for 7,876 samples, 10 cancer related pathways and 32 cancer types in this website (Liu et al., 2018). The “ConsensusClusterPlus” package was used to distinguish different expression patterns based on the mRNA expression data of 51 pyroptosis-related genes. The consensus distributions for each k value were revealed through empirical cumulative distribution function (CDF) plot. We used the cluster consensus plot and CDF plot to confirm the number of clusters and their stability. Then, the TCGA samples were clustered into two clusters and were displayed by t-distributed Stochastic Neighbor Embedding (t-SNE) and heatmap utilizing the “Rtsne” package. Finally, the “survival” package was used to compare the OS of the two clusters using Kaplan-Meier curves with a log-rank test.

### Identification of pyroptosis-related hub genes

The “limma” package was used to obtain the DEGs between the two clusters (C1 vs C2). Determination of DEGs was based on an absolute log2FC of >1 and a *p*-value of 0.05 adjusted by false discovery rate (FDR), which was visualized by the heatmap and volcano map using “pheatmap” and “ggplot2” packages.

Then, the “WGCNA” R package was carried out to identify hub genes. To examine the independence and average connectivity degree of multiple modules with varying power levels, the gradient approach was applied. Among all the soft threshold values, the one that showed the highest mean connectivity was selected (*β* = 2). By adjusting the merging threshold function to 0.25, we finally identified six modules. The first two modules with the highest correlation were chosen (the yellow and turquoise modules). The edges between two genes with weight >0.3 were utilized to form a network based on the genes in yellow and turquoise modules. A total of 410 hub genes were identified for further investigation in the yellow and turquoise modules.

The possible regulatory functions of these genes were discovered using the Gene Ontology (GO) and Kyoto Encyclopedia of Genes and Genomes (KEGG) analysis provided by the R package “clusterProfiler".

### Construction and validation of the prognostic signature

The batch effects between TCGA and GEO datasets were adjusted by empirical Bayes framework with the “sva” R package. Then, univariate Cox regression analysis was carried out to choose prognostic hub genes significantly correlated with OS among the 410 hub genes (yellow and turquoise modules in WGCNA), and 21 genes were selected for further analysis (*p* < 0.01). Next, the 21 genes were used to develop a robust and concise PEPScore model by multivariate Cox regression analysis in TCGA cohort. Consequently, a six-gene PEPScore model was created, with the PEPScore equaling the sum of each patient’s gene expression value (FPKM format) multiplying their coefficients in the multivariate Cox model. Based on the median PEPScore value, every patient in the TCGA and GEO databases was grouped into a high- or low-PEPScore subgroup. Kaplan-Meier survival curves with log-rank were employed to identify the prognostic power of the PEPScore in the two subgroups utilizing the “survival” R package. The co-expression network of the pyroptosis-related genes in two subgroups was conducted through the “igraph” package. The “timeROC” R package was performed to visualize the ROC curves and determine the area under the curves (AUC) for 1-, 3-, and 5-year OS, while the ROC predicting the pyroptosis expression patterns by PEPScore was performed by “pROC” R package. The independent prognostic value of the PEPScore was confirmed using univariate and multivariate Cox regression analysis. Finally, the model performance was compared with other studies through the “survcomp” R package.

### Construction of the nomogram

The nomogram predicting the probability of 1-, 3- and 5- year OS of LUSC was developed by all independent prognostic factors acquired by univariate and multivariate Cox regression analysis. The discrimination performance of the nomogram was assessed by calibration and AUC. The nomogram’s discriminating ability was measured using calibration curve. Then, using the time-ROC curve and decision curve analysis (DCA), we compared the nomogram with all to those with only one independent prognostic factor. The best model is the one with the highest computed net benefit.

### Comprehensive molecular and tumor-microenvironmental profiling in two subgroups

To explore the potential mechanism underlying the difference of PEPScore in different PEPScore groups, we initially used the R package “clusterProfiler” to perform Gene Set Enrichment Analysis (GSEA) on the HALLMARK gene sets. To assess the quality and quantity of gene mutations in two groups, we performed gene mutation analysis and calculated the tumor mutational burden (TMB) using “Maftools” package. The information on genetic alterations was obtained from the TCGA. The patients were dichotomized based on a cut-off of the TMB calculated by R package of “survminer”. According to the cut-off value of TMB (cut-off = 2.105), those with higher TMB were grouped into high-TMB group, and the others were grouped into low-TMB group. The difference in OS between the two groups was assessed using Kaplan-Meier curves with log-rank analysis.

To explore the difference in immune characteristics of 502 LUSC samples, we utilized “CIBERSORT” to assess the relative proportion of 22 different kinds of immune cells. Then we compared the quantity of these cells between high- and low-PEPScore subgroups.

### Exploration of the treatment strategy for two subgroups

The Drugbank database (Wishart et al., 2018) (https://go.drugbank.com/) was used to explore LUSC-related drug target genes. Chemotherapy response of each sample was evaluated by the “pRRophetic” R package (Geeleher et al., 2014) based on Genomics of Drug Sensitivity in Cancer (GDSC), including Cisplatin, Gemcitabine, Docetaxel, Vinblastine, Etoposide and Paclitaxel. To explore the immunotherapy response of each sample, the Tumor Immune Dysfunction and Exclusion (TIDE) algorithm (Fu et al., 2020) (http://tide.dfci.harvard.edu/) was used to determine the TIDE score, TIS score, cell dysfunction score and exclusion score. The radiotherapy sensitivity of each sample was evaluated by the radiosensitivity index (RSI), which was reported in the prior study. (Eschrich et al., 2009).

### Statistical analysis

**FIGURE 1:**
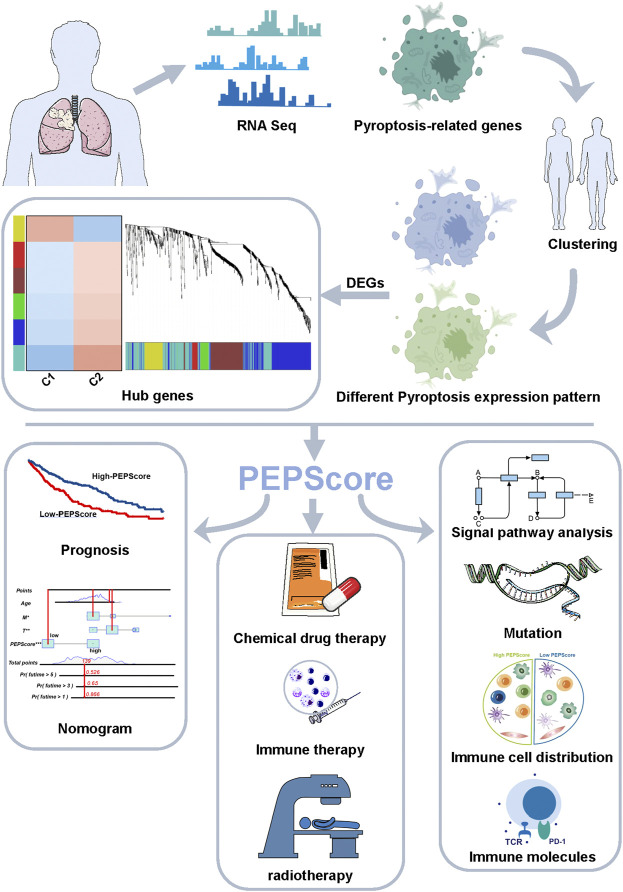
Abstract graphical representation for comprehensive characterization of PEPScore subgroups in LUSC.

Figure 1 depicts the entire analytical procedure. An independent *t*-test was performed to explore the difference of continuous variables with normal distribution between two groups. For continuous variables did not follow a normal distribution, the Wilcoxon test was used. The categorical variables were compared using the Pearson chi-square test. Kaplan-Meier survival analysis with the log-rank test was used for the univariable survival study. Data processing was completed by Perl (version5.30.0) and R software (version 4.1.1). All statistical analyses were conducted with R software. All our codes are available at the github website (https://github.com/chenw265/For_research.git).

## Result

### Classification of pyroptosis-related genes in different expression patterns

**FIGURE 2:**
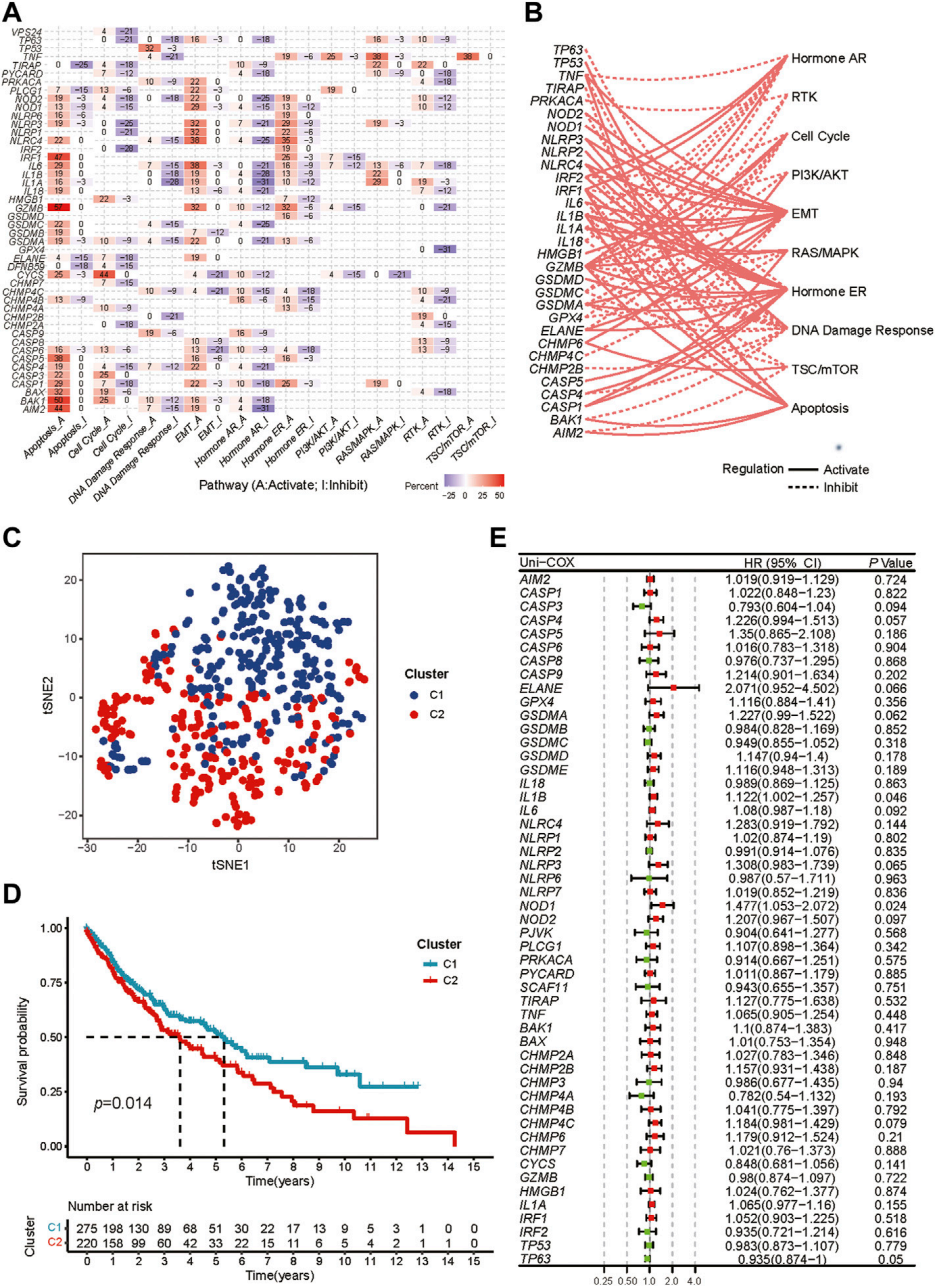
Exploration of two different expression patterns of pyroptosis-related molecules. **(A)** Heatmap depicts the association between pyroptosis-related genes expression levels and essential cancer signaling pathways. The percentage is the total proportion of tumors in which a gene has an influence on the pathway among the 32 cancer types (number of inhibited or activated cancer types/32 *100%). Pyroptosis genes that have a role (inhibit or activate) in at least five cancer types are included in this heatmap. The percentage of tumors in which a pathway may be inhibited by specified genes is represented by “pathway inhibit” (blue), whereas activation is represented by “pathway activate” (red). **(B)** The correlation between the pyroptosis-related genes in LUSC and essential cancer signaling pathways. The dotted line indicates inhibition, whereas the solid line indicates activation. **(C)** t-SNE plot shows two different pyroptosis expression patterns represented by the expression of pyroptosis-related genes. **(D)** Kaplan-Meier curves for the OS between two distinct pyroptosis expression patterns. **(E)** Univariate Cox analysis to explore the prognosis value of each pyroptosis-related genes for LUSC.

42 pyroptosis-related DEGs were identified by differential expression analysis for pyroptosis-related gene expression levels between 502 tumors and 49 normal samples (Supplementary Figure S1A). According to the correlations between the pyroptosis-related genes, majority of them were significantly co-expressed or mutex-expressed. (Supplementary Figure S1B). Then the expression levels of the pyroptosis-related genes in major cancer signaling pathways among 32 cancer types were analyzed though the GSCALite. (Liu et al., 2018). In these tumor tissue, most pyroptosis-related molecules, particularly *IRF1*, *GZMB*, *CASP5*, *BAK1* and *AIM2*, were consistently inhibited in the cell cycle, DNA damage response, hormone AR and RTK signaling pathway, but highly activated in the apoptosis signaling way. (Figure 2A). And in LUSC tissue, most pyroptosis molecules inhibited the hormone AR, cell cycle and DNA damage response pathways but activated the apoptosis, hormone ER and EMT (Figure 2B).

We performed consensus clustering analysis to identify different expression patterns of the pyroptosis-related genes in patients with LUSC. When k = 2, we got the most satisfying Cumulative Distribution Function (CDF), indicating that the LUSC patients could be well grouped into two clusters (Supplementary Figures S2A-H), which was confirmed by heatmap and t-SNE (Supplementary Figure S2I, Figure 2C). The OS between the two expression patterns has a significant difference (Figure 2D).

### Identification of pyroptosis-related hub genes

Preliminary screening for survival-related genes was conducted using univariate Cox regression (Figure 2E). But we found that only two genes (*IL1B* and *NOD1*) met the criteria of *p* < 0.05, which was not satisfactory for the requirements of our model construction.

Therefore, we then analyzed differential expression between two clusters on a genome-wide scale and a total of 962 DEGs were obtained as the candidate genes (n = 962). Heatmap and volcano map for DEGs show obvious differences (Supplementary Figures S3A, B). Candidate genes were analyzed by WGCNA analysis and six modules were identified using the optimal soft-thresholding power and the average linkage hierarchical clustering. (Supplementary Figure S3C, Supplementary Figure S4). A total of 410 genes in the turquoise and yellow modules were further chosen as hub genes, whose expression patterns are most closely related with the two different pyroptosis status. The gene network in turquoise and yellow modules was displayed in Supplementary Figure S3D. According to GO analysis, the hub genes were enriched in cell differentiation and immune-related process. According to KEGG analysis, the hub genes were mainly correlated with *staphylococcus aureus* infection, hematopoietic cell lineage, rheumatoid arthritis, cytokine-cytokine receptor interaction, *etc.* (Supplementary Figure S3E, detailed in Supplementary Table S2).

### Construction and validation of the prognostic model

**FIGURE 3:**
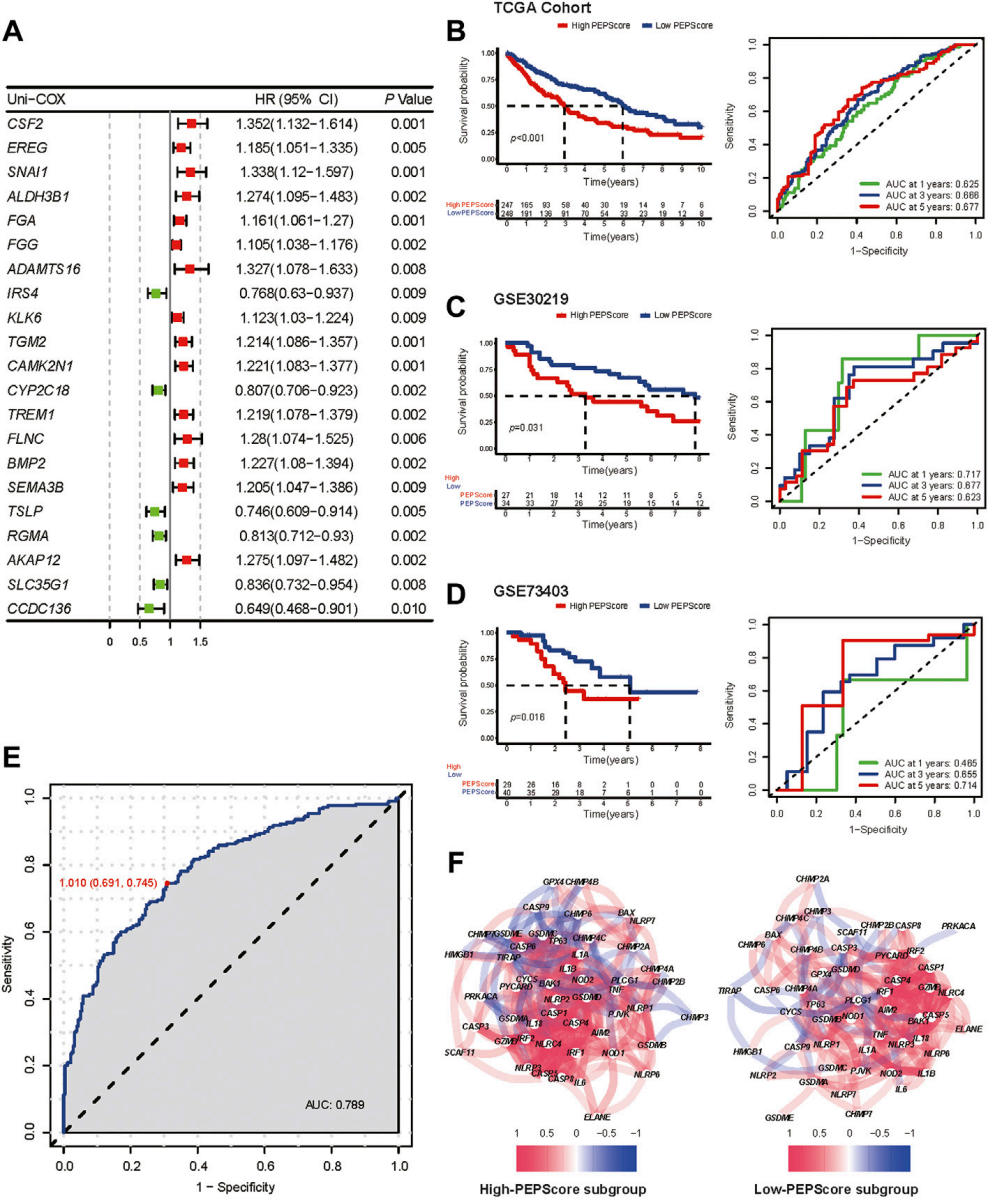
Prognostic value and the characteristics of different PEPScore subgroups. **(A)** The forest plot depicts the result of univariate Cox analysis on 21 pyroptosis-related hub genes. **(B)** Kaplan-Meier survival analysis and ROC curves for patients in the TCGA cohort to identify the prognostic power of the PEPScore. **(C and D)** Kaplan-Meier survival analysis and ROC curves for patients in the GSE30219 and GSE73403 cohort to validate the prognostic power of the PEPScore. **(E)** ROC curve showing the specificity and sensitivity for PEPScore to predict the pyroptosis expression patterns. **(F)** The co-expression network of the pyroptosis-related genes in the high-PEPScore subgroup and low-PEPScore subgroup.

In order to identified the genes that are highly correlated with OS among the hub genes whose expression patterns are most closely related with the two different pyroptosis status, the univariate Cox regression analysis was carried out among the selected 410 hub genes (yellow and turquoise modules in WGCNA), and 21 genes were identified (Figure 3A). Six genes (*CSF2*, *FGA*, *IRS4*, *CYP2C18*, *TSLP*, *AKAP*12) correlated with prognosis were further identified by multivariate cox regression analysis among these 21 genes. We used these genes to develop a pyroptosis-related prognostic model named Pyroptosis Expression Pattern Score (PEPScore). The PEPScore was calculated as follows: PEPScore=expression level ofCSF2×0.18+expressionlevelofFGA×0.09+expressionlevel ofAKAP12×0.41+expression levelofIRS4×(−0.20)+expression level of CYP2C18×(−0.11)+expression level of TSLP×(−0.19). The coefficient of the formula is obtained from multivariate Cox regression analysis, while the expression level of genes is in FPKM format (Supplementary Table S3). Each patient was grouped into low-PEPScore and high-PEPScore subgroups based on the median value of the PEPScore.

As demonstrated by Kaplan-Meier curves, the high-PEPScore subgroup had a lower survival probability than the low-PEPScore subgroup. The ROC curve further confirmed that the PEPScore had good prediction ability and the AUC was 0.625 for 1-year, 0.666 for 3-year and 0.677 for 5-year OS (Figure 3B). Two GEO datasets were applied for external validation. A significant difference in OS was found between the low-PEPScore and high-PEPScore subgroups according to Kaplan-Meier curves, which was consistent with the result of TCGA data. ROC curve indicated that the PEPScore possessed an excellent predictive efficacy as well (Figures 3C, D).

Furthermore, the ROC curve indicated that PEPScore had the best specificity of 0.691 and the best sensitivity of 0.745 to predict different pyroptosis expression patterns (C1, C2) when the median value of the PEPScore was the cut-off value of the ROC curve (Figure 3E). The pyroptosis-related gene co-expression network and the pyroptosis-related gene expression levels between the subgroups were significantly different, which suggested that the PEPScore and pyroptosis were closely related (Figure 3F, Supplementary Figure S5A). Besides, there is significant co-expression or mutex-expression between six model genes and most pyroptosis-related genes (Supplementary Figure S5B).

Finally, although we did not use pyroptosis-related genes to construct model directly in a common way, the PEPScore still shows great prediction accuracy. Li et al. directly used pyroptosis-related genes as input and construct a nine-gene risk model using LASSO in LUSC, and their risk model also shows good performance. (Li et al., 2022).However, the AUC of the PEPScore was higher than Li et al.’s risk model in 1-, three- and 5-year OS. The C-index of PEPScore was also higher than Li et al.’s risk model’s as well (Supplementary Figure S6).

### Clinical characteristics of the PEPScore

Univariate and multivariate Cox regression analysis were used to validate the independent prognostic value of PEPScore (Supplementary Figure S7A). Additionally, the traditional clinical characteristics were not statistically different except for gender. (Supplementary Figure S7B).

To extend the clinical applicability of PEPScore, we developed a nomogram in the TCGA cohort by integrating clinical variables (Supplementary Figure S7C). Each patient obtained a total score based on a combination of the points for prognostic criteria. Patients with a higher total score had a worse prognostic effect. The calibration plot shows that the nomogram acted consistently with an ideal model (Supplementary Figure S7D). Decision curve analysis (DCA) and ROC curve analysis demonstrated that prediction specificity of the nomogram was the best, followed by PEPScore, age, or TNM staging (Supplementary Figure S7E, F).

### Comprehensive analysis of molecular and tumor-microenvironmental characteristics in subgroups

**FIGURE 4:**
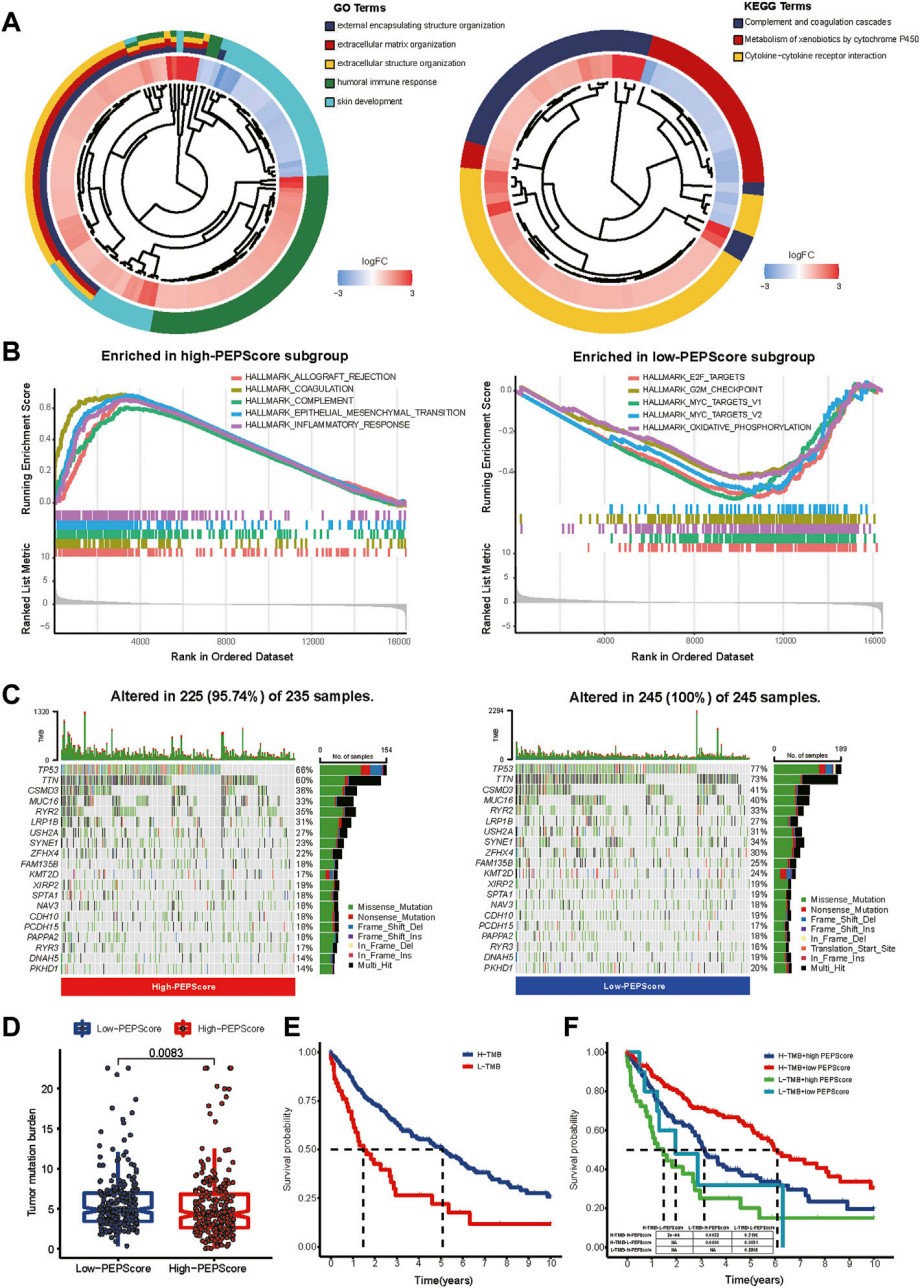
Comprehensive analysis of molecular and tumor-microenvironmental characteristics in PEPScore subgroups. **(A)** GO and KEGG analysis for revealing the potential regulatory mechanisms underlying the difference of PEPScore in different subgroups. A total of 821 DEGs were obtained from differential expression analysis between high- and low-PEPScore subgroups. **(B)** GSEA used on the HALLMARK gene sets to explore the potential mechanism underlying the difference of PEPScore in different subgroups. **(C)** Top 20 mutated molecules in the LUSC patients in TCGA database of different PEPScore subgroups. Each column represents an individual and the mutated genes are arranged by mutation frequency. The color block indicates mutation type, the number on the right shows the mutation percentage, and the figure above shows the TMB. **(D)** TMB calculation to access the quality and quantity of gene mutations in two PEPScore subgroups. **(E)** The Kaplan-Meier curves with the log-rank test show significant differences in OS between high and low TMB subgroups. The cut-off value of TMB was 2.105, which was calculated by R package of “survminer”. **(F)** The Kaplan-Meier curves with the log-rank test show significant differences in OS among LUSC patients with different PEPScore and TMB.

According to the GO and KEGG analysis, DEGs obtained from the differential expression analysis between the high-PEPScore and low-PEPScore subgroups (a total of 821 DEGs) were mainly enriched in immunological and cell differentiation signaling pathways (Figure 4A, detailed in Supplementary Table S4). GSEA showed that the gene sets of low-PEPScore were mainly correlated with tumor proliferation signaling pathways, while the gene sets of high-PEPScore were mainly correlated with tumor metastasis and immune response signaling pathways. (Figure 4B, detailed in Supplementary Table S5).

To further understand the PEPScore, we then analyzed gene mutations between the subgroups. High-PEPScore subgroup had a lower mutation rate than low-PEPScore subgroup, most of which were missense mutations. *TP53* mutation was the most common mutation, followed by *TTN* mutation in both high-PEPScore and low-PEPScore subgroups (Figure 4C). Then we analyzed the mutation of the PEPScore model genes. *FGA* and *IRS4* had the highest mutation rates, accounting for 3%. And missense mutation accounted for the largest part (Supplementary Figure S8A).

And then, we analyzed the relationship between PEPScore and the TMB. The difference analysis showed that the high-PEPScore group got a lower TMB (Figure 4D *p* = 0.0083). The high-TMB group had a clear survival advantage over the low-TMB group (Figure 4E). And the Kaplan-Meier curves illuminate those patients with low TMB and high PEPScore got the shortest median OS, while those with high TMB and low PEPScore got the longest one (Figure 4F, *p* < 0.001).

**FIGURE 5:**
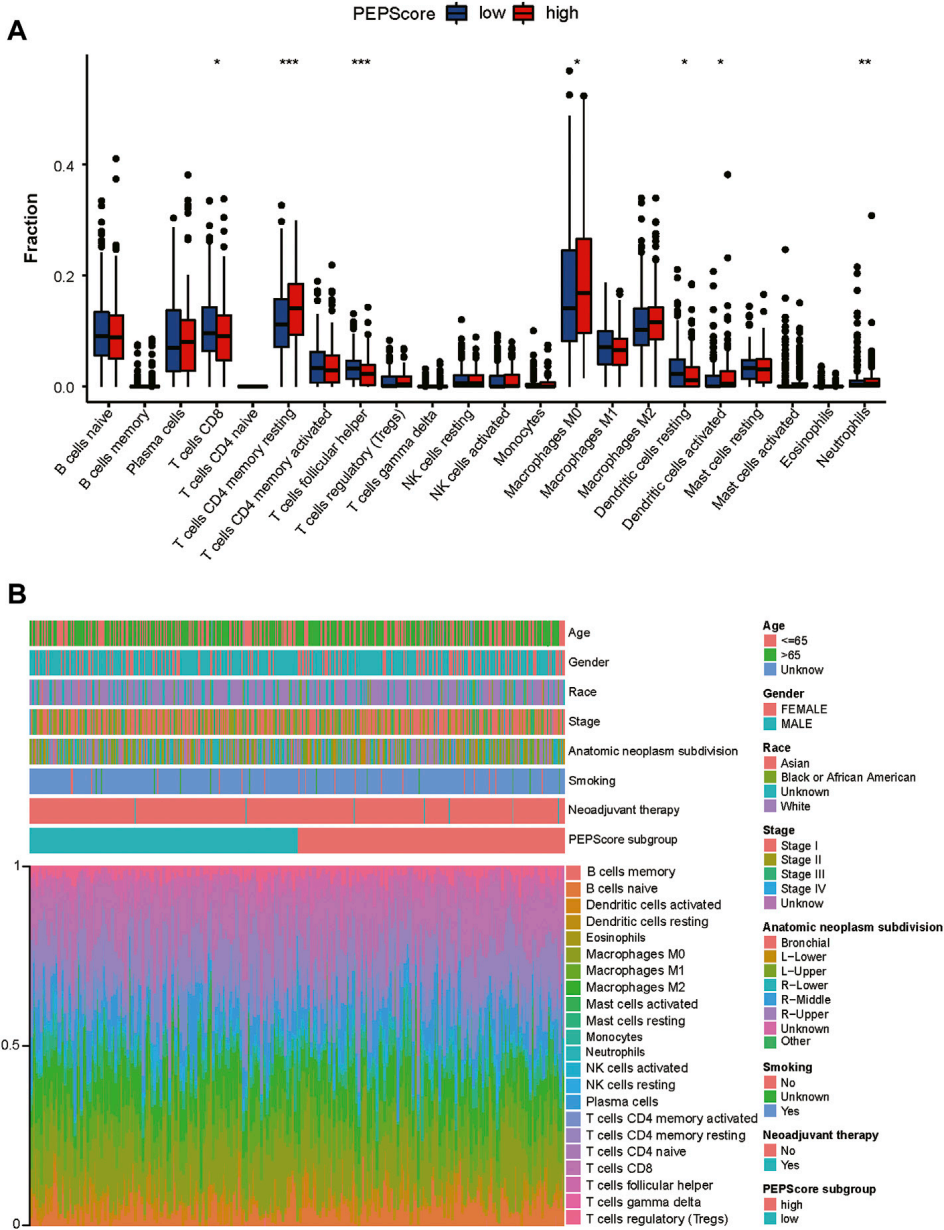
The landscape of the TME and the characteristics of different PEPScore subgroups. **(A)** The proportions of immune cells in the two PEPScore subgroups. The thick line in the box indicates the median value, whereas the dispersed dots indicate an outlier. The upper and bottom border of the box reflects the 25th and 75th percentiles. Asterisk denotes the *p*-value (*: *p* < 0.05, **: *p* < 0.01, and ***: *p* < 0.001). **(B)** PEPScore categorization and TEM cell proportions for 495 patients in the TCGA dataset. Patient annotations include gender, stage, race, age, smoking, and neoadjuvant treatment.

Then infiltration of immune cells was analyzed through “CIBERSORT” and was compared between PEPScore subgroups by the Wilcoxon test. There are more abundant T cells CD4 memory resting, macrophages M0, dendritic cells activated and neutrophils in the high-PEPScore subgroup, while there are more abundant T cells CD8, T cells follicular helper and dendritic cells resting in the low-PEPScore subgroup (Figure 5A). Characteristics correlated with the immune landscape, which includes the clinicopathological characteristics of different PEPScore subgroups, are shown in Figure 5B. According to the correlation analysis between immune cells and the six model genes, *AKAP*12 and *CSF2* showed a negative correlation with the infiltration of T follicular helper cells and CD8 T cells, and they were also positively correlated with neutrophils, T cells CD4 memory resting, *etc.* Especially, *CSF2* was the gene that had a significantly strong correlation with most immune cells (Supplementary Figure S8B).

Then we explored the relationship between PEPScore and the checkpoint molecules and chemokine receptors expression levels. We found that PEPScore was significantly positively correlated with the expression levels of the checkpoint molecules and chemokine receptors, except for *VTCN1*, while the association between *CD274* and *IDO1* was statistically insignificant (Supplementary Figure S9).

### The role of PEPScore subgroups in clinical therapy

**FIGURE 6:**
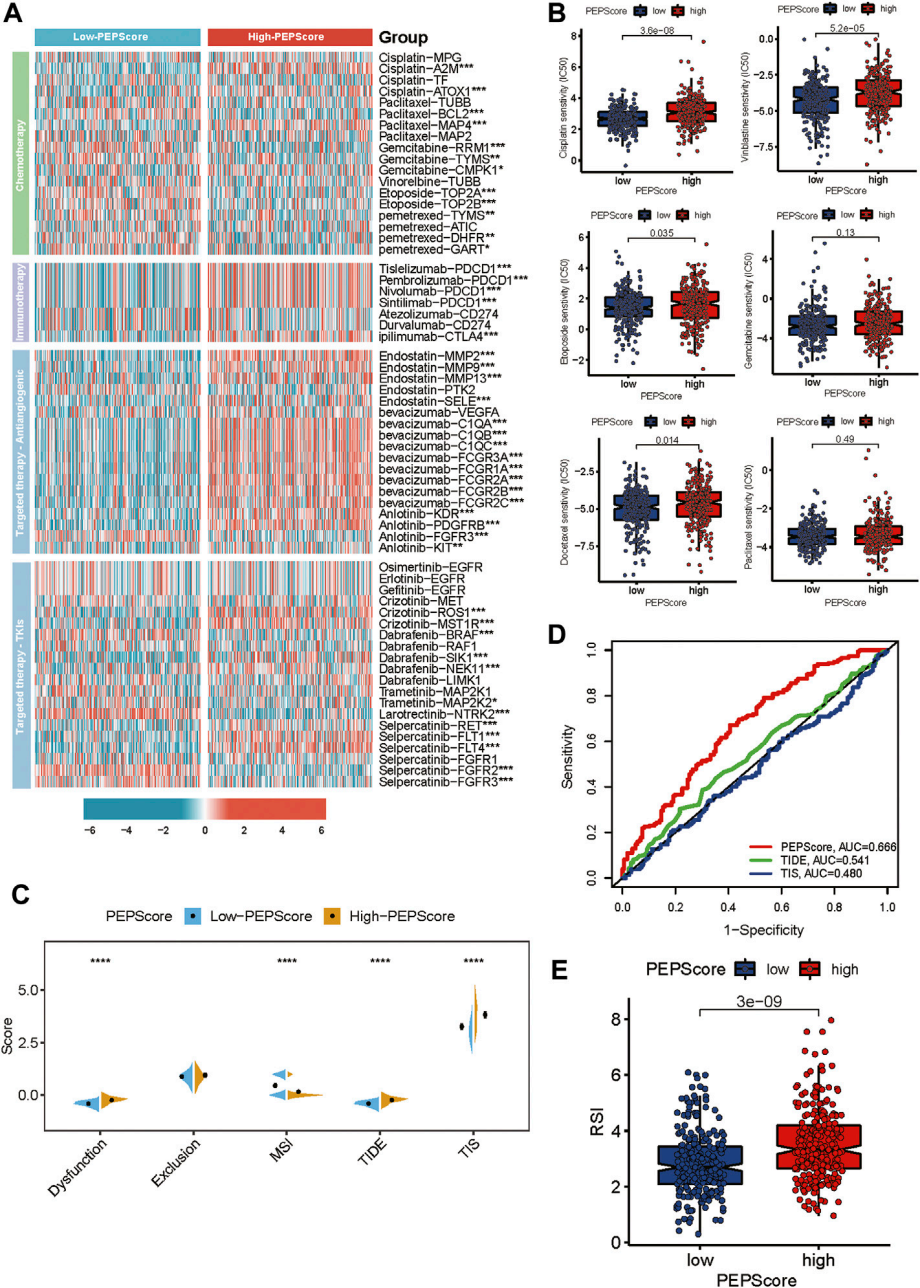
PEPScore predicts drug sensitivity. **(A)** The heatmap presents the different expressions of common drug targets for LUSC patients in high-PEPScore and low-PEPScore subgroups. Asterisk denotes the *p*-value (*: *p* < 0.05, **: *p* < 0.01, and ***: *p* < 0.001). **(B)** The difference in IC50 of the common chemotherapeutic drugs between high- and low-PEPScore subgroups. **(C)** The Wilcoxon test shows the difference in TIDE, MSI, TIS and T cell exclusion and dysfunction scores in high- and low-PEPScore subgroups. The *p*-value is indicated by asterisk (****p < 0.0001). **(D)** ROC curve analysis of the predictive value of the PEPScore, TIDE and TIS. **(E)** The difference in Radiotherapy index (RSI) between high- and low-PEPScore subgroups.

We investigated the relationship between PEPScore and the clinical efficacy of LUSC therapy. We analyzed the expression differences of common chemotherapeutic drug targets in LUSC between the subgroups, including drugs of chemotherapy, immune checkpoint inhibitors, antiangiogenic drugs and tyrosine kinase inhibitors. We found that the expression level of the Tislelizumab, Pembrolizumab, Nivolumab and Sintilimab target (*PDCD1*), ipilimumab target (*CTLA4*), Bevacizumab targets (*C1QA*, *C1AB*, *C1QC*, *FCGR3A*, *FCGR1A*, *FCGR2A*, *FCGR2B* and *FCGR2C*), Anlotinib targets (*KDR*, *PDFGRB*, *FGFR3* and *KIT*) and Crizotinib (*ROS1*, *MST1R*) were higher in high-PEPScore subgroup. While the expression level of target genes for Gemcitabine, Etoposide, and Larotrectinib were higher in low-PEPScore subgroup (Figure 6A). Besides, we used “pRRophetic” R tools to calculate the IC50 value of drugs and we found that the IC50 of Cisplatin, Vinblastine, Etoposide and Docetaxel was obviously lower in the low-PEPScore subgroup, implying a negative association between the chemotherapeutic drug sensitivity of LUSC and PEPScore (Figure 6B). TIDE is a computational framework developed to evaluate the potential of tumor immune escape from gene expression, serving as a surrogate biomarker to evaluate the response to immune checkpoint blockade. According to the TIDE algorithm, the TIDE score in the low-PEPScore subgroup was found to be lower than the high-PEPScore subgroup, which suggested that low-PEPScore patients might benefit more from immunotherapy. And MIS score was higher in the low-PEPScore subgroup, while the T cell dysfunction score as well as TIS score were higher in high-PEPScore subgroup (Figure 6C). The predictive value of PEPScore was estimated by ROC curves. We found that the AUC of PEPScore was better than TIDE and TIS, indicating that the predictive value of PEPScore was as excellent as TIDE and TIS for OS (Figure 6D). On top of these two kinds of therapies, we also explored the relationship between radiotherapy and PEPScore. The low-PEPScore subgroup got a lower RSI score than high-PEPScore subgroup, suggesting that the high-PEPScore subgroup was less expected to benefit from radiotherapy (Figure 6E).

## Discussion

In this study, we first analyzed differential expression of 51 pyroptosis-related genes in tumor and non-tumor tissues, as well as the association between these pyroptosis-related genes and cancer signaling pathways. We found that most of them were different and associated with various cancer signaling pathways. Based on pyroptosis-related DEGs, two pyroptosis expression patterns with different prognosis were identified through consensus clustering. Nevertheless, the association between pyroptosis-related gene expression and LUSC patient prognosis was not satisfactory enough in univariate Cox analysis. This may be caused by the mutual compensation of the complex signaling pathway network in humans. Thus, we identified the DEGs between different pyroptotic expression patterns on the whole genome, and we used WGCNA combined with univariate cox analysis to identify 21 pyroptosis expression pattern hub genes and established prognostic model PEPScore based on six genes (*CSF2*, *FGA*, *AKAP*12, *CYP2C18*, *IRS4*, *TSLP*). PEPScore was shown to be a reliable prognostic pyroptosis-related biomarker for LUSC. High PEPScore suggested better survival while low PEPScore was the opposite in both TCGA and GEO cohorts. Besides, ROC and DCA showed that combining PEPScore with conventional clinical prognostic factors could better predict patients’ OS.

PEPScore was made of six genes, *CSF2*, *FGA*, *AKAP*12, *CYP2C18*, *IRS4*, and *TSLP*. Colony-stimulating factor 2 (*CSF2*, also known as *GM-CSF*), secreted as monomeric glycoproteins, can control the production, differentiation, and function of granulocytes and macrophages. (Ingelfinger et al., 2021). *CSF2* could induce pyroptosis-related molecule expression in the neutrophils, including IL-1B, caspase-1 (p20) and NLRP3. (Furuya et al., 2018). Although a few studies believe that *CSF2* inhibits tumor progression, most studies have shown that it can stimulate various types of tumor cell growth and migration, including lung cancer, gliomas and skin carcinoma. (Dong et al., 2012; Hong, 2016). Thymic stromal lymphopoietin (*TSLP*), an IL-7-like inflammatory factor could promote TH2 cell responses that are involved in immunity in various inflammatory diseases. High expression of *TSLP* could up-regulate the expression of GSDMD-N, IL-1beta, as well as IL-18 in human THP-1macrophages, inducing Caspase-1-dependent pyroptosis through activation of NLRP3 inflammasome. (Moon and Kim, 2011; Ji et al., 2021). Indeterminately, in certain studies, *TSLP* has a cancer-promoting effect, whereas in others, a cancer-protective effect. (Dong et al., 2012). We found that lower expression of *TSLP* led to a poorer prognosis, providing some insights for further studies. Fibrinogen alpha chain (*FGA*) polymerizes with FGB and FGG to form an insoluble fibrin matrix, which is an extracellular matrix protein participating in blood clot formation as well as tumor angiogenesis and metastasis. A-kinase anchoring protein 12 (*AKAP*12) is a member of the *AKAP* protein kinase family that suppresses tumors. The expression of *AKAP*12 is down-regulated in various cancers including colon cancer, childhood acute lymphoblastic leukemia and hepatocellular carcinoma, *etc.* Insulin receptor substrate 4 (*IRS4*), a cytoplasmic protein containing many potential phosphorylation sites, is overexpressed in NSCLC. Cytochrome P450 family 2 subfamily C member 18 (*CYP2C18*), is a member of the superfamily of cytochrome P450 enzymes, which are monooxygenases involved in drug metabolism and other substances. It is reported to be correlated with esophageal cancer, gastric adenocarcinoma and breast cancer. Although our results demonstrate that pyroptosis-related genes expression and the six model genes have various degrees of association, the relationship between the *FGA*, *AKAP*12, *CYP2C18* and *IRS4* and the pyroptosis remains unclear. From the calculation formula of PEPScroe, we found that the *CSF2*, *FGA* and *AKAP*12 and PEPScore were positively correlated, while *CYP2C18*, *IRS4* and *TSLP* and PEPScore were negatively correlated. In conclusion, all these six genes are significantly involved in pyroptosis and cancer development, which may be a potential therapeutic target.

Although we did not use pyroptosis-related genes to establish models directly like most studies, the PEPScore still shows a strong association with pyroptosis (Ye et al., 2021; Chen et al., 2022; Yang et al., 2022; Yu et al., 2022). The ROC curve shows high specificity and sensitivity for PEPScore to distinguish different pyroptotic expression patterns. Moreover, the expression of the pyroptosis-related genes and their correlations are significantly different between the PEPScore subgroups. The way our model constructed is an entirely different approach from previous studies, and our results also demonstrate the reliability of this method. It is worth mentioning that the model constructed by our method has a better performance than the model constructed by common method used on a previous study in LUSC (Li et al., 2022).

To further acquire the biological insight into the PEPScore, we explored and compared gene mutation between the PEPScore subgroups. The most frequent mutation is missense mutation, followed by nonsense mutation and frameshift deletions. The most common mutation gene in both groups, *TP53*, is more frequent in low-PEPScore subgroup, as reported previously. Although *TP53* is a tumor suppressor gene, mutation of *TP53* can significantly upregulate the expression of interferon-gamma, activated T-effector and immune checkpoint, which indicates more likely to benefit from PD-1 inhibitors. Besides, the second most frequently mutated gene between two subgroups was *TTN*, which is considered associated with TMB, and high *TTN* mutation is revealed to be related to better survival. (Yang et al., 2020). Therefore, high-PEPScore LUSC patients with low *TP53* and *TTN* mutation possess a worse prognosis compared with low-PEPScore LUSC patients with high *TP53* and *TTN* mutations.

Different from apoptosis, pyroptosis can provoke different degrees of inflammation reaction and is considered related to immunity. (Liu et al., 2021). Our GO, KEGG and GSEA analysis also suggested that pyroptosis can affect the tumor immune microenvironment. Therefore, further understanding of the TMB and the landscape of the TME can provide a more complete understanding of the biological characteristics of PEPScore as well as provide guidance for finding a new therapeutic regimen for LUSC or improving immunotherapy effect. TMB is a potential biomarker to predict ICI therapy efficacy. (Yarchoan et al., 2017). In our study, patients with high TMB and low PEPScore had significantly better prognosis compared with patients with low TMB and high PEPScore, and in the same PEPScore subgroup, patients with high TMB had better prognosis compared with patients with low TMB, suggesting that TMB can help explain why PEPScore influence the immunotherapy effect. But not explaining all of it, there may still be other mechanisms. Besides, the infiltration of the immune cells in two PEPScore subgroups is different. Neutrophils and M0 macrophages were enriched in high-PEPScore subgroup, while T follicular helper cells (TFH), cytotoxic CD8 T cells as well as dendritic resting cells were more abundant in low-PEPScore subgroup. Previous results revealed that high density of the T cell infiltration, especially cytotoxic CD8 T cells, indicating a better prognosis. (Gentles et al., 2015). The presence of the TFH, which is critical for the germinal center formation and gives necessary help for B cell mutation and function, is considered related to prolonging survival in most human cancers. Neutrophils are also regarded as tumor accomplices since they can regulate tumor survival and migration, angiogenesis as well as immune response, promoting tumor progression and metastasis. (Xiao et al., 2021). Our results support these conclusions. The different components of the immune cells in different PEPScore subgroups may result from the different pyroptosis states of the tumor cells, which has a different regulation effect on tumor immune microenvironment. Based on the correlation analysis between model genes and immune cells, the expression of *AKAP*12 and *CSF2* has a negative correlation with the infiltration of CD8 T cells as well as T follicular helper cells, which may be because the expression of these genes promotes these cells undergoing pyroptosis, leading to poor prognosis in LUSC patients.

Finally, we confirm that PEPScore is reliable in predicting the prognosis of patients with LUSC as well as providing guidance on therapy selection. Our results show that different types of chemotherapeutic drug targets were expressed at different levels in PEPScore subgroups. Moreover, TIDE and MSI, considered effective biomarkers for immunotherapy, are also different in different PEPScore subgroups. (Jiang et al., 2018). Interestingly, despite the high expression of immunotherapy targets in the high-PEPScore subgroup, their TIDE was low, which is inconsistent with the previous report that up-regulated immunotherapy targets are correlated with better immunotherapy effects. We speculate that this may be because of the aforementioned changes in the pyroptotic state of the cancer cells, which affects their immune microenvironment and promotes tumor immune escape. Regrettably, the subgroup analysis of the IC50 of chemotherapeutic drugs, immunotherapy TIDE score and RSI suggests that any single treatment method is not effective enough for the high-PEPScore subgroup, and they may need combination therapy.

Although our multidimensional results show that the PEPScore has great predict effects in LUSC, this study still had some limitations that need to be considered. Firstly, our study results cannot provide the exact mechanism by which pyroptosis modulates the prognosis in LUSC. Some experiments for exploring the potential mechanism are needed. Secondly, this study cannot explain the exact mechanism by which model genes of PEPScore affect the LUSC pyroptosis status. Therefore, in subsequent studies, further exploration of the specific mechanisms by which model genes alter the pyroptosis status of tumor cells is necessary. Moreover, a large-scale clinical cohort validation is still lacking before the PEPScore enter into the application in clinical practice. These have not only increased the challenges but also provided us with optimism, making us more motivated to continue digging.

In conclusion, we constructed a PEPScore model which was validated internally and externally to predict the prognosis of LUSC patients. PEPScore is correlated with gene mutation and tumor immune microenvironment in terms of molecular biological function. The PEPScore overall performance on the validated datasets shows that the model is robust with broad application prospects.

## Data Availability

The datasets analyzed for this study can be found in the TCGA database at https://portal.gdc.cancer.gov/. R codes used for analysis in this study were are accessible *via* Github Page at: https://github.com/chenw265/For_research.git.

## References

[B1] Chen J. Tao Q. Lang Z. Gao Y. Jin Y. Li X. (2022). Signature construction and molecular subtype identification based on pyroptosis-related genes for better prediction of prognosis in hepatocellular carcinoma. Oxid. Med. Cell. Longev. 2022, 4494713. 10.1155/2022/4494713 35069975 PMC8767411

[B2] Dong J. Hu Z. Wu C. Guo H. Zhou B. Lv J. (2012). Association analyses identify multiple new lung cancer susceptibility loci and their interactions with smoking in the Chinese population. Nat. Genet. 44 (8), 895–899. 10.1038/ng.2351 22797725 PMC6628171

[B3] Eschrich S. A. Pramana J. Zhang H. Zhao H. Boulware D. Lee J. H. (2009). A gene expression model of intrinsic tumor radiosensitivity: Prediction of response and prognosis after chemoradiation. Int. J. Radiat. Oncol. Biol. Phys. 75 (2), 489–496. 10.1016/j.ijrobp.2009.06.014 19735873 PMC3038688

[B4] Fu J. Li K. Zhang W. Wan C. Zhang J. Jiang P. (2020). Large-scale public data reuse to model immunotherapy response and resistance. Genome Med. 12 (1), 21. 10.1186/s13073-020-0721-z 32102694 PMC7045518

[B5] Furuya M. Y. Asano T. Sumichika Y. Sato S. Kobayashi H. Watanabe H. (2018). Tofacitinib inhibits granulocyte-macrophage colony-stimulating factor-induced NLRP3 inflammasome activation in human neutrophils. Arthritis Res. Ther. 20 (1), 196. 10.1186/s13075-018-1685-x 30157949 PMC6116484

[B6] Gao J. Qiu X. Xi G. Liu H. Zhang F. Lv T. (2018). Downregulation of GSDMD attenuates tumor proliferation via the intrinsic mitochondrial apoptotic pathway and inhibition of EGFR/Akt signaling and predicts a good prognosis in non-small cell lung cancer. Oncol. Rep. 40 (4), 1971–1984. 10.3892/or.2018.6634 30106450 PMC6111570

[B7] Geeleher P. Cox N. Huang R. S. (2014). pRRophetic: an R package for prediction of clinical chemotherapeutic response from tumor gene expression levels. PLoS One 9 (9), e107468. 10.1371/journal.pone.0107468 25229481 PMC4167990

[B8] Gentles A. J. Newman A. M. Liu C. L. Bratman S. V. Feng W. Kim D. (2015). The prognostic landscape of genes and infiltrating immune cells across human cancers. Nat. Med. 21 (8), 938–945. 10.1038/nm.3909 26193342 PMC4852857

[B9] Hong I. S. (2016). Stimulatory versus suppressive effects of GM-CSF on tumor progression in multiple cancer types. Exp. Mol. Med. 48 (7), e242. 10.1038/emm.2016.64 27364892 PMC4973317

[B10] Ingelfinger F. De Feo D. Becher B. G. (2021). GM-CSF: Master regulator of the T cell-phagocyte interface during inflammation. Semin. Immunol. 54, 101518. 10.1016/j.smim.2021.101518 34763973

[B11] Ji Q. Wang L. Liu J. Wu Y. Lv H. Wen Y. (2021). Aspergillus fumigatus-stimulated human corneal epithelial cells induce pyroptosis of THP-1 macrophages by secreting TSLP. Inflammation 44 (2), 682–692. 10.1007/s10753-020-01367-x 33118609

[B12] Jiang A. Meng J. Bao Y. Wang A. Gong W. Gan X. (2021). Establishment of a prognosis prediction model based on pyroptosis-related Signatures associated with the immune microenvironment and molecular heterogeneity in clear cell renal cell carcinoma. Front. Oncol. 11, 755212. 10.3389/fonc.2021.755212 34804944 PMC8603037

[B13] Jiang P. Gu S. Pan D. Fu J. Sahu A. Hu X. (2018). Signatures of T cell dysfunction and exclusion predict cancer immunotherapy response. Nat. Med. 24 (10), 1550–1558. 10.1038/s41591-018-0136-1 30127393 PMC6487502

[B14] Li T. Liu H. Dong C. Lyu J. (2022). Prognostic implications of pyroptosis-related gene Signatures in lung squamous cell carcinoma. Front. Pharmacol. 13, 806995. 10.3389/fphar.2022.806995 35153782 PMC8829032

[B15] Li X. Y. Zhang L. Y. Li X. Y. Yang X. T. Su L. X. (2021). A pyroptosis-related gene signature for predicting survival in glioblastoma. Front. Oncol. 11, 697198. 10.3389/fonc.2021.697198 34485134 PMC8416108

[B16] Liberzon A. Birger C. Thorvaldsdóttir H. Ghandi M. Mesirov J. P. Tamayo P. (2015). The Molecular Signatures Database (MSigDB) hallmark gene set collection. Cell. Syst. 1 (6), 417–425. 10.1016/j.cels.2015.12.004 26771021 PMC4707969

[B17] Liu C. J. Hu F. F. Xia M. X. Han L. Zhang Q. Guo A. Y. (2018). GSCALite: A web server for gene set cancer analysis. Bioinformatics 34 (21), 3771–3772. 10.1093/bioinformatics/bty411 29790900

[B18] Liu X. Xia S. Zhang Z. Wu H. Lieberman J. (2021). Channelling inflammation: Gasdermins in physiology and disease. Nat. Rev. Drug Discov. 20 (5), 384–405. 10.1038/s41573-021-00154-z 33692549 PMC7944254

[B19] Moon P. D. Kim H. M. (2011). Thymic stromal lymphopoietin is expressed and produced by caspase-1/NF-κB pathway in mast cells. Cytokine 54 (3), 239–243. 10.1016/j.cyto.2011.03.007 21463955

[B20] Moussette S. Al Tuwaijri A. Kohan-Ghadr H. R. Elzein S. Farias R. Berube J. (2017). Role of DNA methylation in expression control of the IKZF3-GSDMA region in human epithelial cells. PLoS One 12 (2), e0172707. 10.1371/journal.pone.0172707 28241063 PMC5328393

[B21] Papi A. Casoni G. Caramori G. Guzzinati I. Boschetto P. Ravenna F. (2004). COPD increases the risk of squamous histological subtype in smokers who develop non-small cell lung carcinoma. Thorax 59 (8), 679–681. 10.1136/thx.2003.018291 15282388 PMC1747095

[B22] Shi J. Gao W. Shao F. (2017). Pyroptosis: Gasdermin-Mediated programmed necrotic cell death. Trends biochem. Sci. 42 (4), 245–254. 10.1016/j.tibs.2016.10.004 27932073

[B23] Siegel R. L. Miller K. D. Fuchs H. E. Jemal A. (2022). Cancer statistics, 2016. Ca. Cancer J. Clin. 72 (1), 7–30. 10.3322/caac.21332 35020204

[B24] Subramanian A. Tamayo P. Mootha V. K. Mukherjee S. Ebert B. L. Gillette M. A. (2005). Gene set enrichment analysis: A knowledge-based approach for interpreting genome-wide expression profiles. Proc. Natl. Acad. Sci. U. S. A. 102 (43), 15545–15550. 10.1073/pnas.0506580102 16199517 PMC1239896

[B25] Wishart D. S. Feunang Y. D. Guo A. C. Lo E. J. Marcu A. Grant J. R. (2018). DrugBank 5.0: A major update to the DrugBank database for 2018. Nucleic Acids Res. 46 (D1), D1074–D1082. 10.1093/nar/gkx1037 29126136 PMC5753335

[B26] Xiao Y. Cong M. Li J. He D. Wu Q. Tian P. (2021). Cathepsin C promotes breast cancer lung metastasis by modulating neutrophil infiltration and neutrophil extracellular trap formation. Cancer Cell. 39 (3), 423–437. e7. 10.1016/j.ccell.2020.12.012 33450198

[B27] Yang Y. Zhang J. Chen Y. Xu R. Zhao Q. Guo W. (2020). MUC4, MUC16, and TTN genes mutation correlated with prognosis, and predicted tumor mutation burden and immunotherapy efficacy in gastric cancer and pan-cancer. Clin. Transl. Med. 10 (4), e155. 10.1002/ctm2.155 32898332 PMC7443139

[B28] Yang Z. Chen Z. Wang Y. Wang Z. Zhang D. Yue X. (2022). A novel defined pyroptosis-related gene signature for predicting prognosis and treatment of glioma. Front. Oncol. 12, 717926. 10.3389/fonc.2022.717926 35433410 PMC9008739

[B29] Yarchoan M. Hopkins A. Jaffee E. M. (2017). Tumor mutational burden and response rate to PD-1 inhibition. N. Engl. J. Med. 377 (25), 2500–2501. 10.1056/NEJMc1713444 29262275 PMC6549688

[B30] Ye Y. Dai Q. Qi H. (2021). A novel defined pyroptosis-related gene signature for predicting the prognosis of ovarian cancer. Cell. Death Discov. 7 (1), 71. 10.1038/s41420-021-00451-x 33828074 PMC8026591

[B31] Yu H. Fu Y. Tang Z. Jiang L. Qu C. Li H. (2022). A novel pyroptosis-related signature predicts prognosis and response to treatment in breast carcinoma. Aging (Albany NY) 14 (2), 989–1013. 10.18632/aging.203855 35085103 PMC8833126

[B32] Zhang T. Li Y. Zhu R. Song P. Wei Y. Liang T. (2019). Transcription factor p53 suppresses tumor growth by prompting pyroptosis in non-small-cell lung cancer. Oxid. Med. Cell. Longev. 2019, 8746895. 10.1155/2019/8746895 31737176 PMC6815571

