# Supplementary material for: A pyroptosis expression pattern score predicts prognosis and immune microenvironment of lung squamous cell carcinoma
Source: Front Genet. 2022 Nov 10;13:996444. doi: 10.3389/fgene.2022.996444 (PMC9685532; doi:10.3389/fgene.2022.996444)
Supplement: Supplementary file 3 [file DataSheet1.zip › Supplementary Figures/Description of Supplementary Figures.docx]

**Supplementary Figure**

**Supplementary Figure 1. Expression characteristics of the pyroptosis-related genes in LUSC.**

(A) Differential expression analysis between 502 tumors and 49 normal samples. Asterisk denotes the P-value (*: P < 0.05, **: P < 0.01, and ***: P < 0.001). (B) Expression characteristic of the pyroptosis-related genes in 502 patients with LUSC in the TCGA. The red color denotes co-expression, the blue color denotes mutex-expression, and the asterisk denotes a P-value of less than 0.05.

**Supplementary Figure 2. Consensus clustering of the LUSC patients based on the expression of the pyroptosis-related genes.**

(A-H) heatmaps of the consistency matrix with k=2 to k=9. (I) Empirical cumulative distribution function (CDF) plot for evaluating consensus distributions of each k value. (J) CDF delta area curve of consensus clustering from 2 to 9 of k

**Supplementary Figure 3. Identification of pyroptosis-related hub genes.**

(A) Heatmap showing the differential expression genes (DEGs) between the two pyroptosis expression patterns (C1 vs. C2) with P-value < 0.05 and |log2FC| > 1. (B) Volcano map for DEGs between the two pyroptosis expression patterns (C1 vs. C2). (C) Gene modules grouped by WGCNA. (D) The gene network in yellow and turquoise modules. (E) Gene Ontology (GO) and Kyoto Encyclopedia of Genes and Genomes (KEGG) analysis for revealing the potential regulatory mechanisms. A total of 410 hub genes were obtained from the yellow and turquoise modules in WGCNA.

**Supplementary Figure 4. Determination of the soft-thresholding power in the WGCNA analysis.**

(A) Network topology analysis for different soft-thresholding powers. (B) Hierarchical clustering tree showing each module.

**Supplementary Figure 5. The correlation between the pyroptosis-related gene expression and the PEPScore model.**

(A) Boxplot shows the differential expression of the pyroptosis-related genes in different PEPScore subgroups. P-value is indicated as asterisk (*: P < 0.05, **: P < 0.01, and ***: P < 0.001). (B) Heatmap displaying the correlation between six PEPScore model genes and the pyroptosis-related genes. P-value is indicated as asterisk (*: P < 0.05, **: P < 0.01, and ***: P < 0.001).

**Supplementary Figure 6. Comparation of the accuracy of PEPScore with previous study.**

(A) Kaplan-Meier survival analysis and ROC curves for patients in the TCGA cohort to identify the prognostic power of the risk model in Li et al. study. (B) the C-index of the PEPScore and the risk model in Li et al. study.

**Supplementary Figure 7. Clinical significance of the PEPScore.**

(A) Univariate Cox analysis of clinical factors and the PEPScore, and multivariate Cox analysis of the factors significant in the univariate Cox analysis (P < 0.05). (B) Wilcoxon test of PEPScore variation in age, gender, stage and race. (C) A nomogram for evaluating prognostic effect in the TCGA dataset. (D) Nomogram calibration plots for estimating the OS at 1, 3, and 5 years in the TCGA dataset. (E) Decision curve analysis (DCA) for nomogram, PEPScore, age, gender and stage. (F) ROC curve analysis of the prediction value of nomogram, PEPScore, age and stage.

**Supplementary Figure 8. Comprehensive analysis of molecular characteristics of the six PEPScore model genes.**

(A) Gene mutation analysis on the six PEPScore model genes. Each column represents an individual and the model genes are arranged by mutation frequency. The color block indicates mutation type, the illustration above depicts the TMB, the number on the right depicts the mutation percentage, and the figure at the bottom indicates the clinical characteristics. (B) Heatmap displaying the association between the six PEPScore model genes and the immune cells. P-value is indicated as asterisk (*: P < 0.05, **: P < 0.01, and ***: P < 0.001).

**Supplementary Figure 9. The correlation between PEPScore and the checkpoint molecules and chemokine receptors expression level.**
