# Supplementary figures and images for: A pyroptosis expression pattern score predicts prognosis and immune microenvironment of lung squamous cell carcinoma
Source: Front Genet. 2022 Nov 10;13:996444. doi: 10.3389/fgene.2022.996444 (PMC9685532; doi:10.3389/fgene.2022.996444)

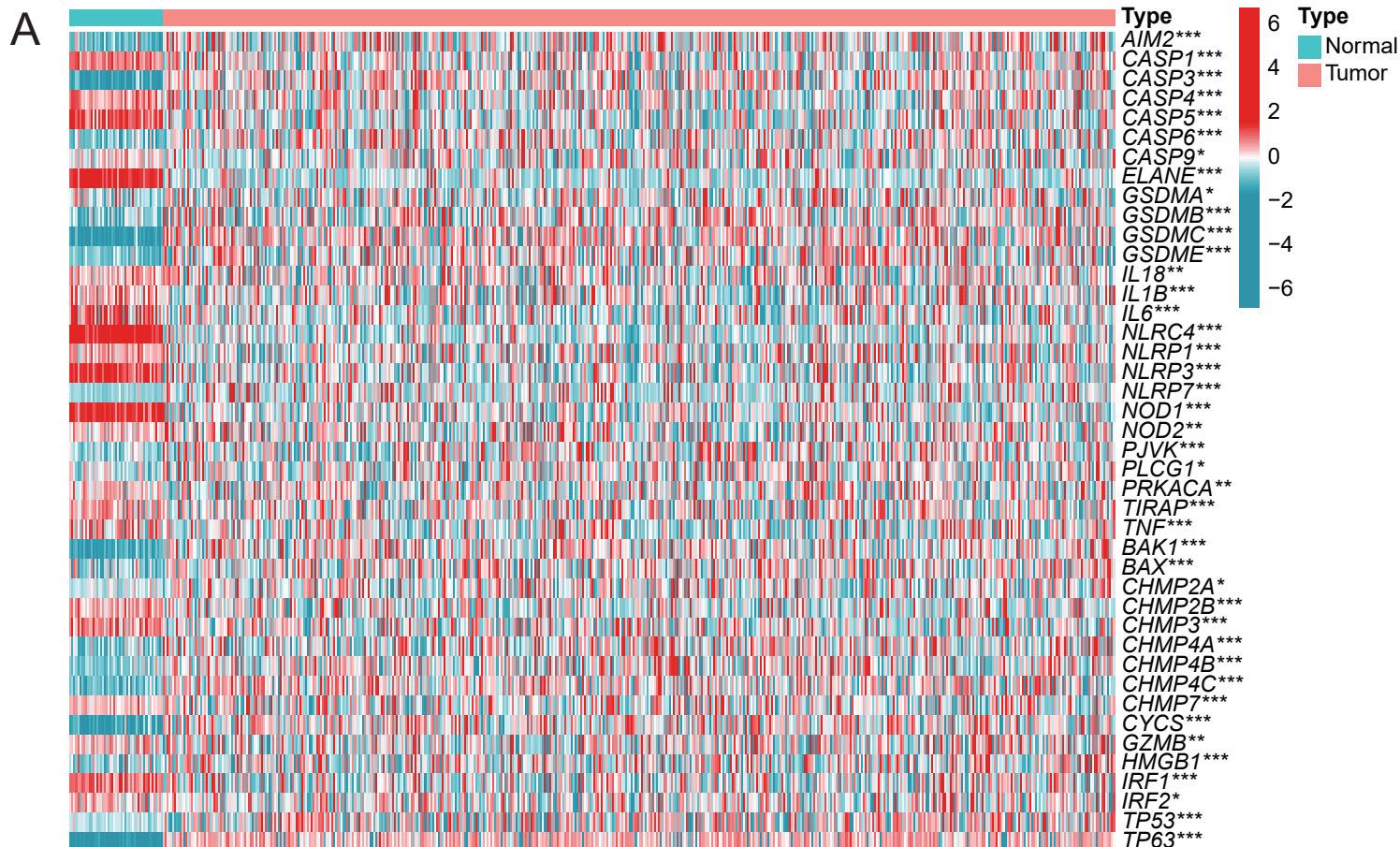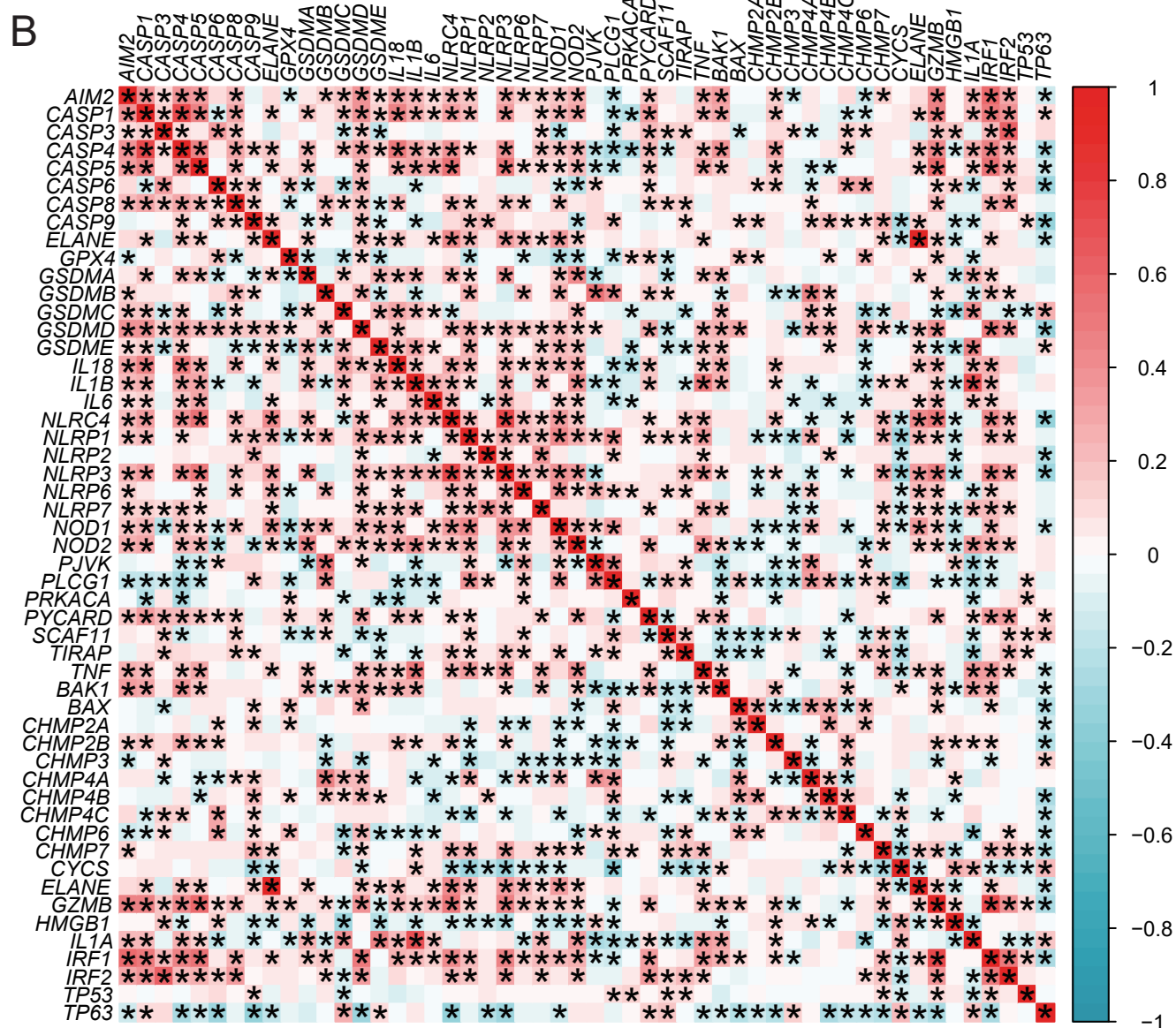

Supplement: Supplementary file 3 [file DataSheet1.zip › Supplementary Figures/S1.pdf]

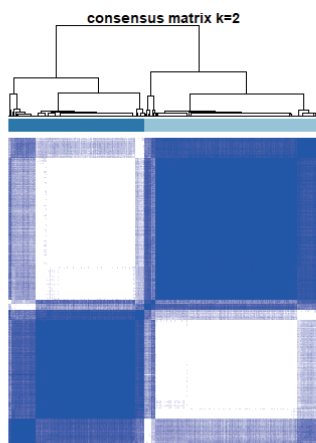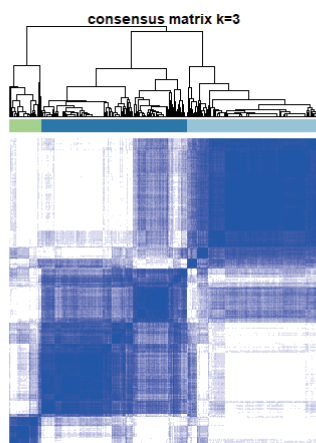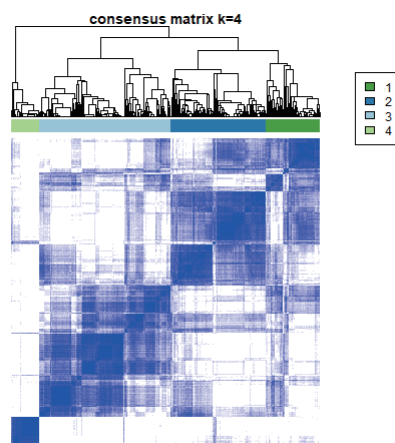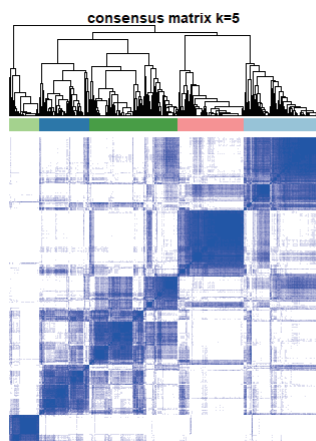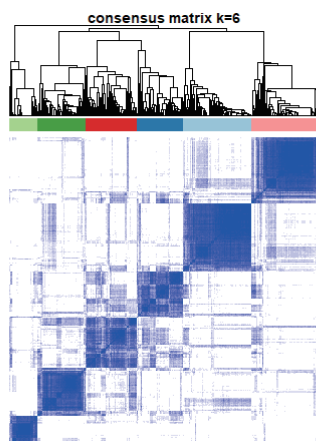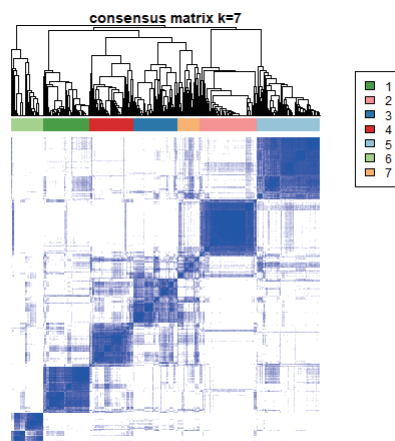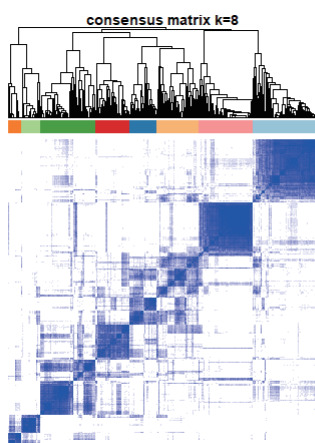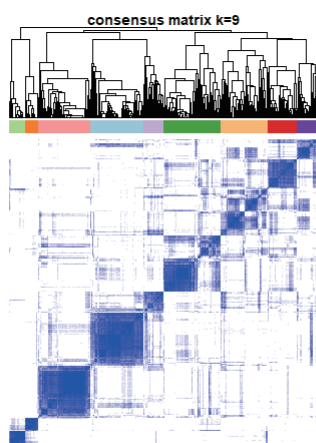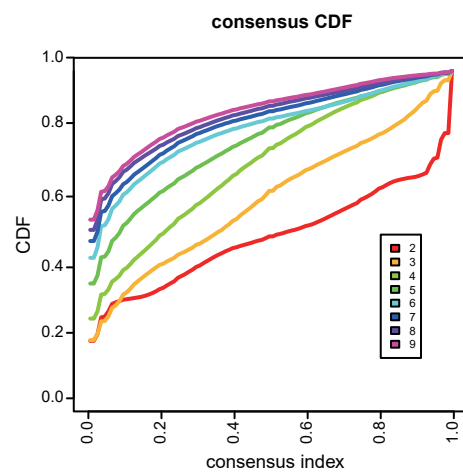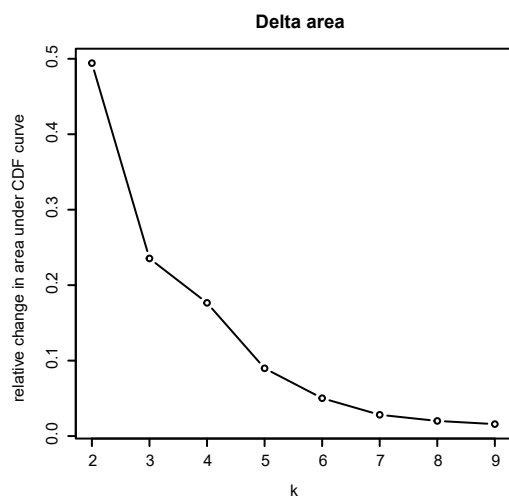

Supplement: Supplementary file 3 [file DataSheet1.zip › Supplementary Figures/S2.pdf]

A

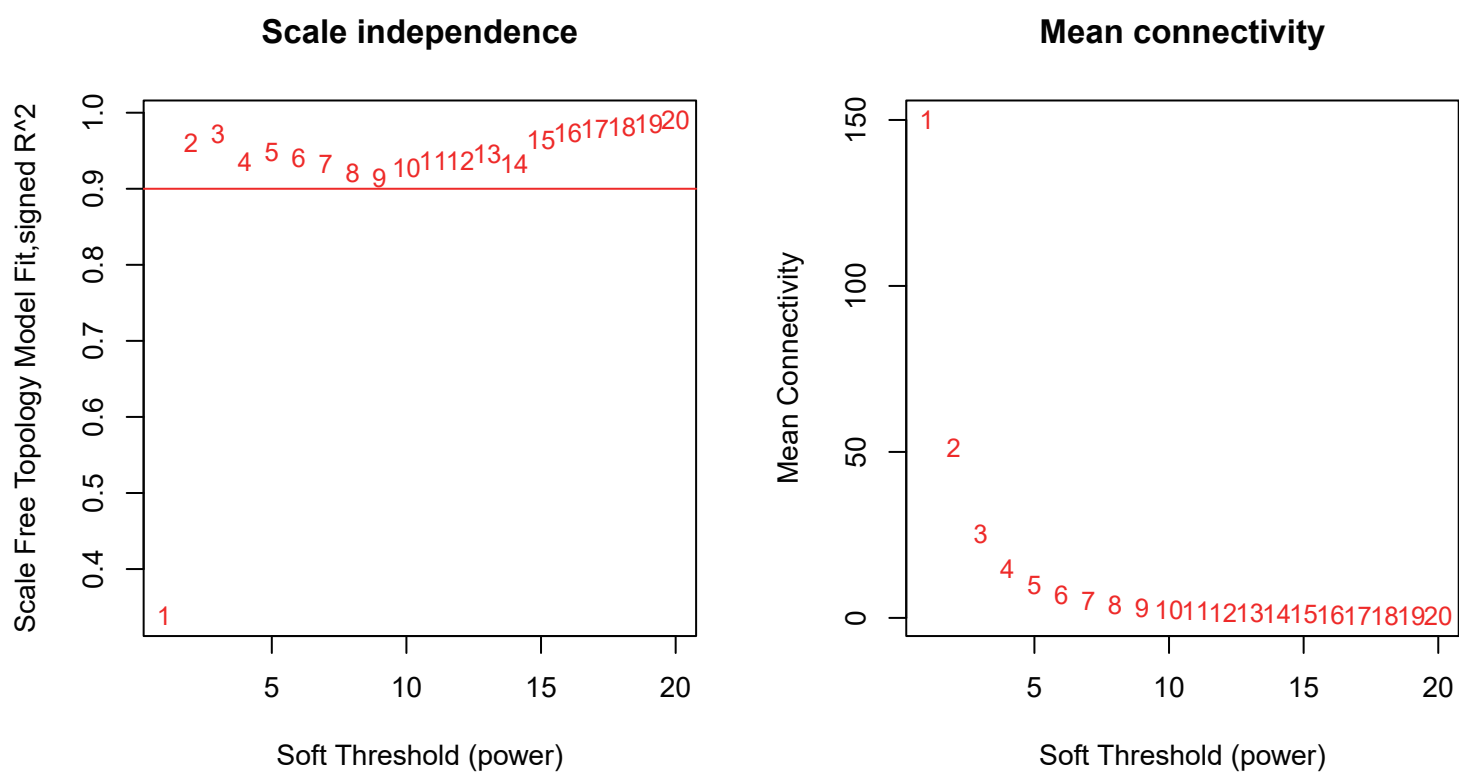

B

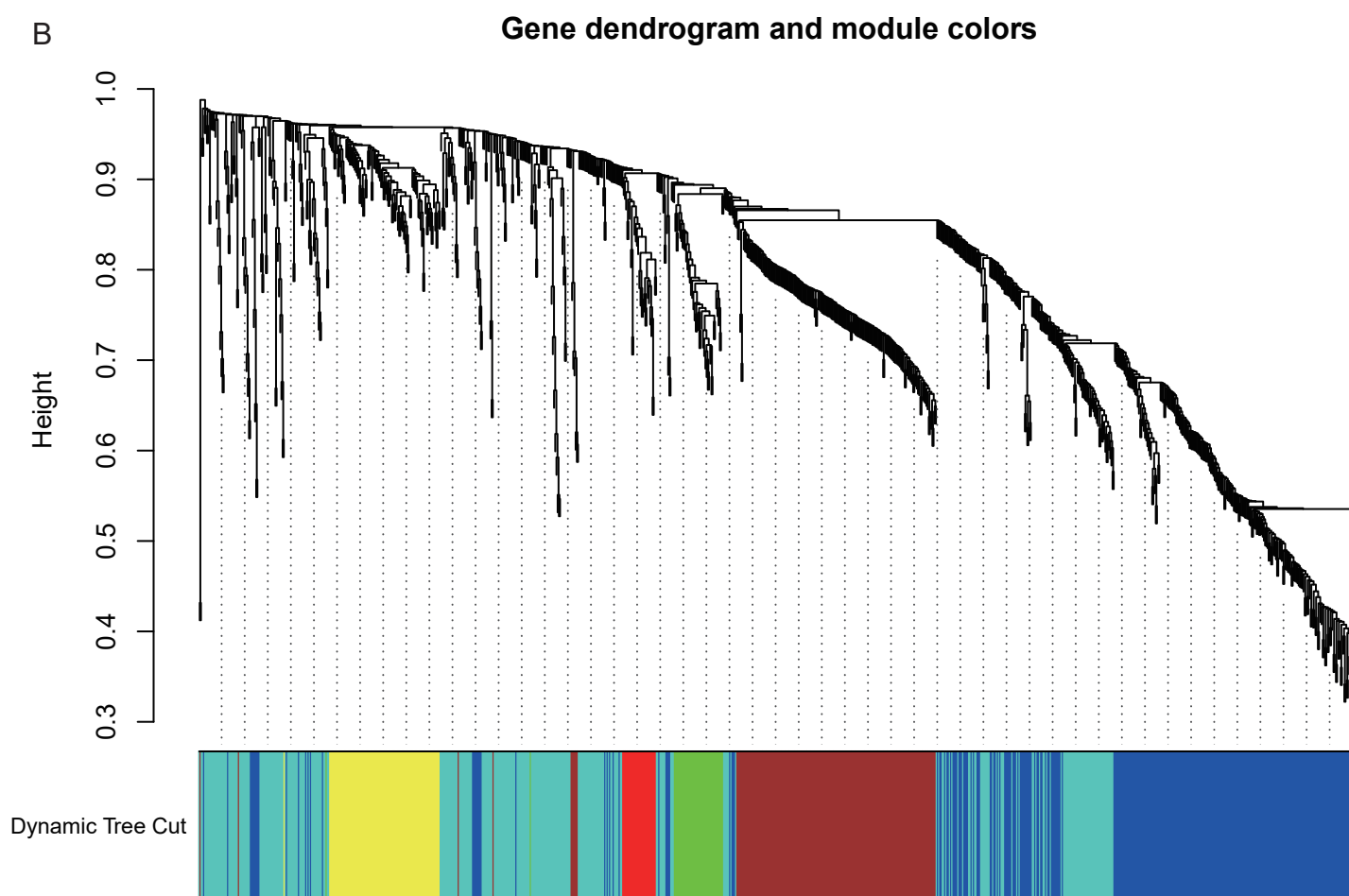

Supplement: Supplementary file 3 [file DataSheet1.zip › Supplementary Figures/S4.pdf]

A

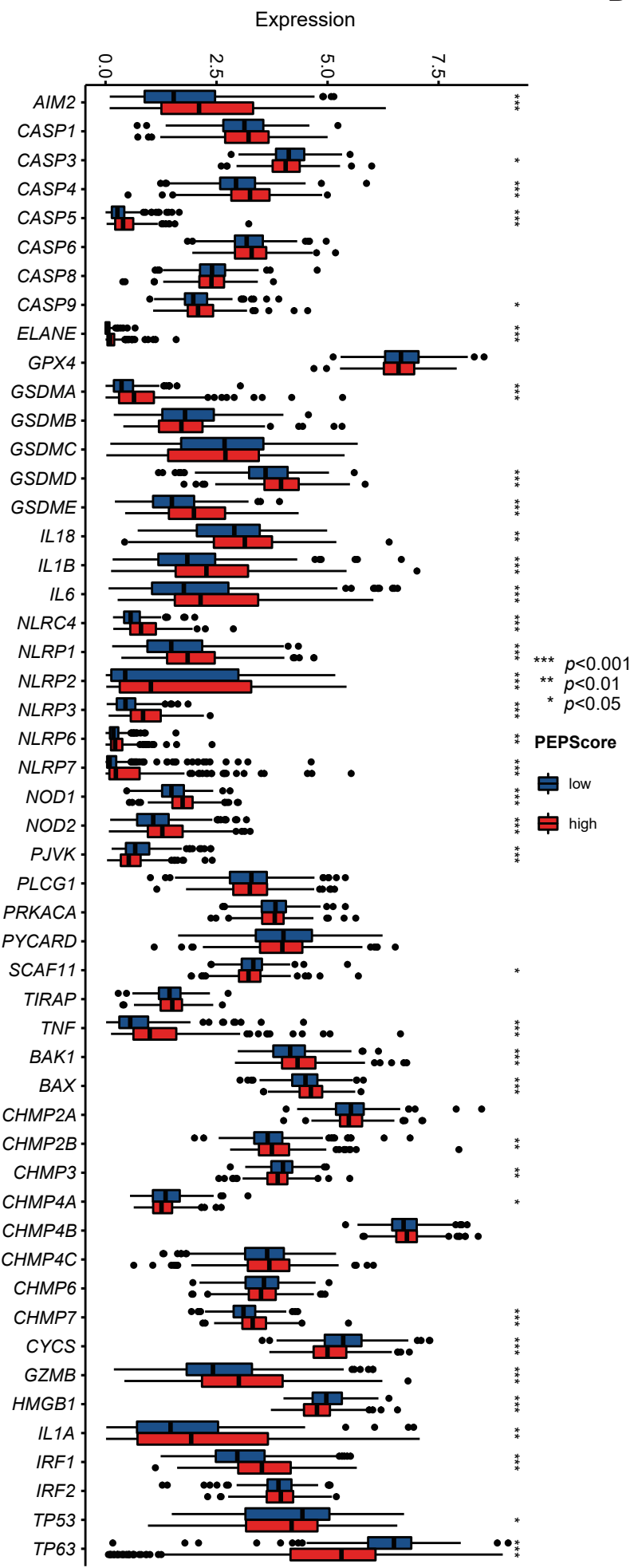

B

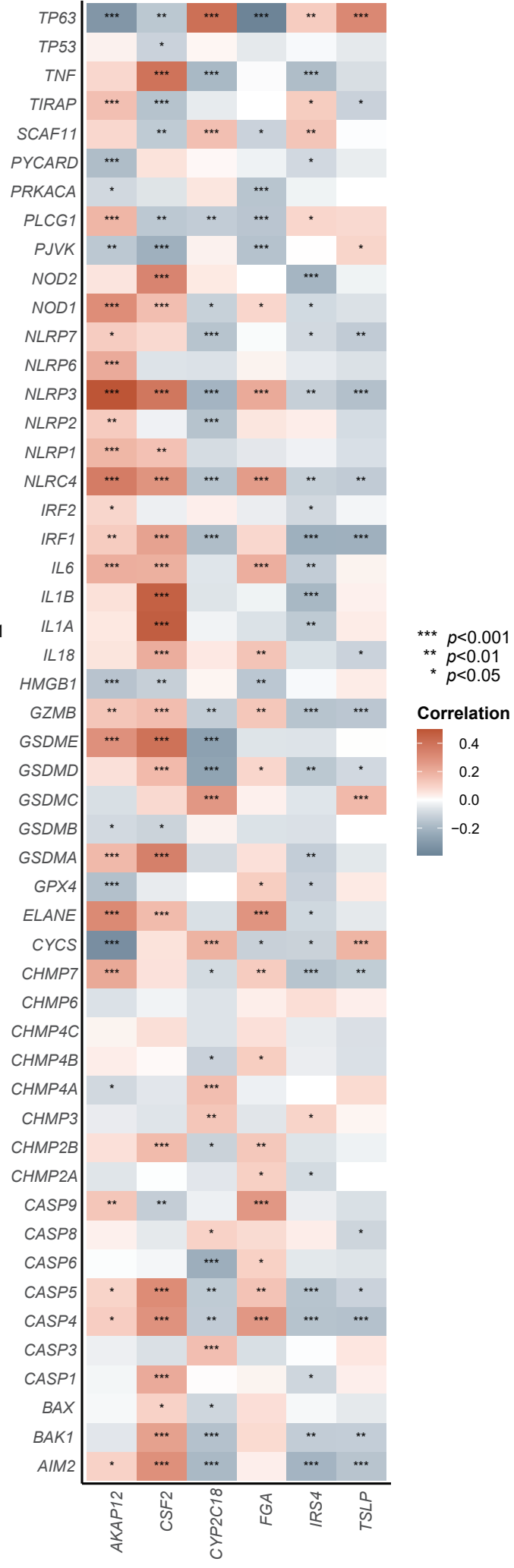

Supplement: Supplementary file 3 [file DataSheet1.zip › Supplementary Figures/S5.pdf]

A

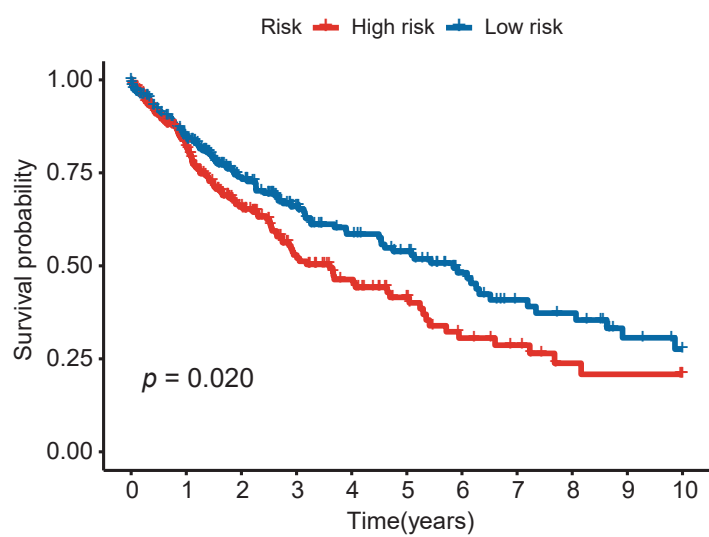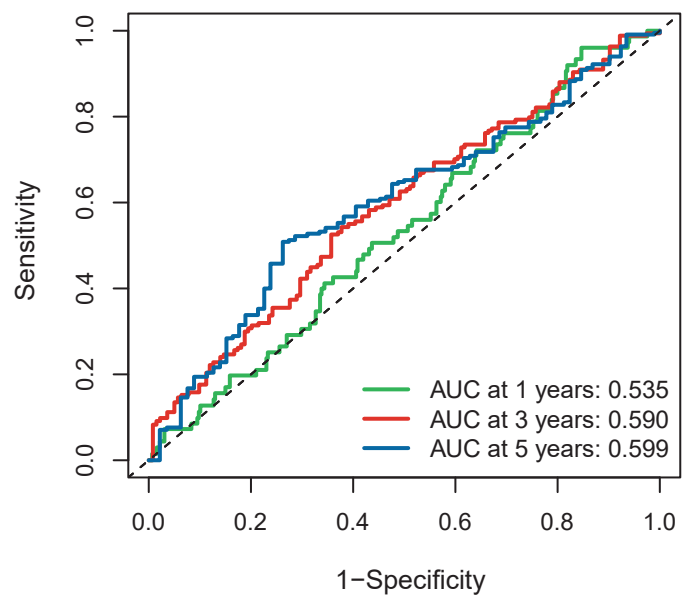

B

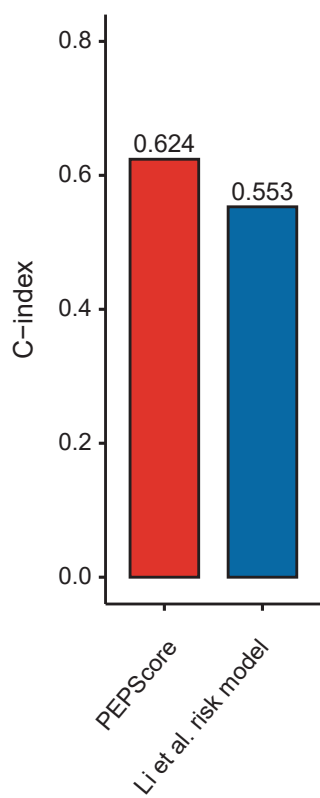

Supplement: Supplementary file 3 [file DataSheet1.zip › Supplementary Figures/S6.pdf]

A

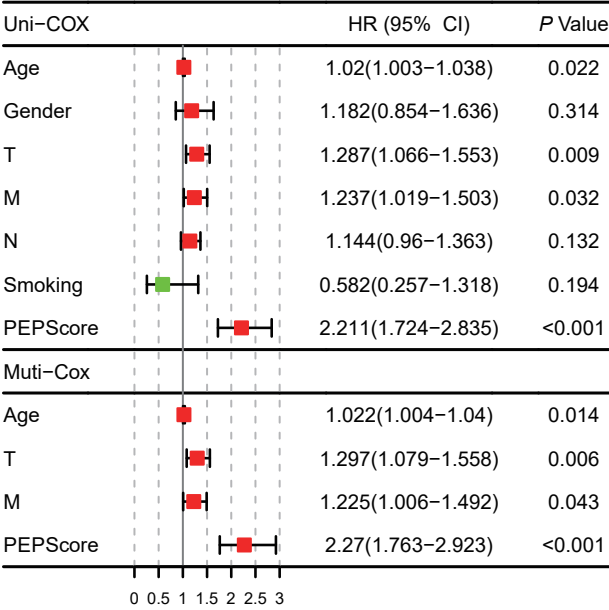

B

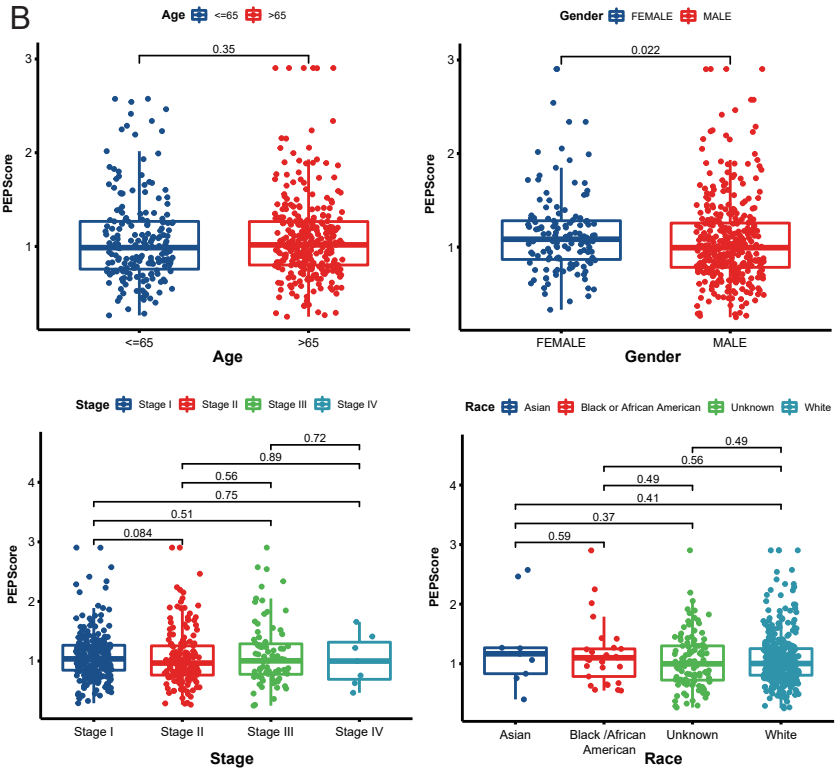

C

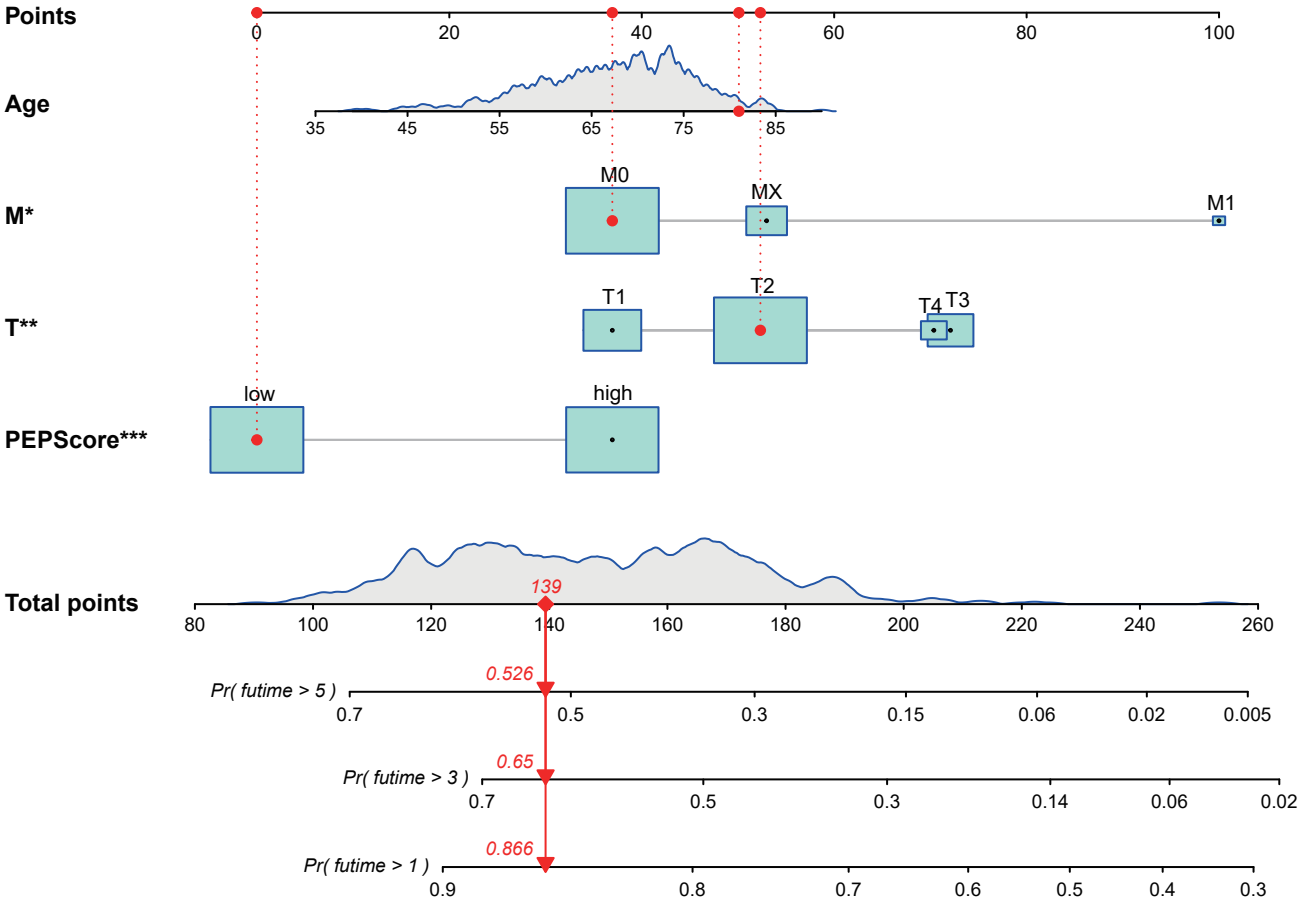

D

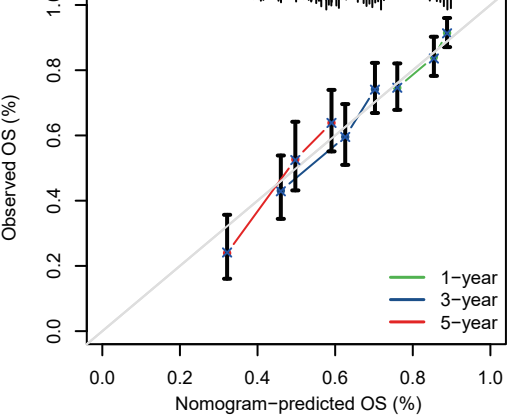

E

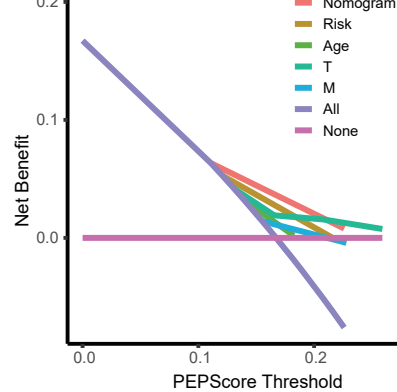

F

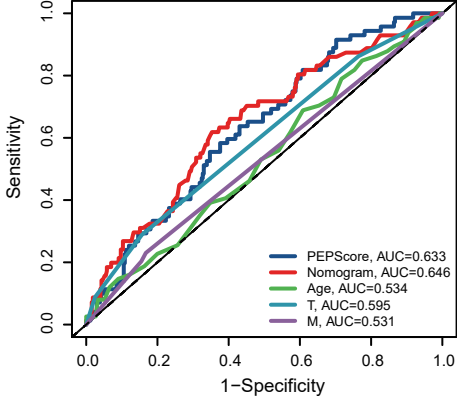

Supplement: Supplementary file 3 [file DataSheet1.zip › Supplementary Figures/S7.pdf]

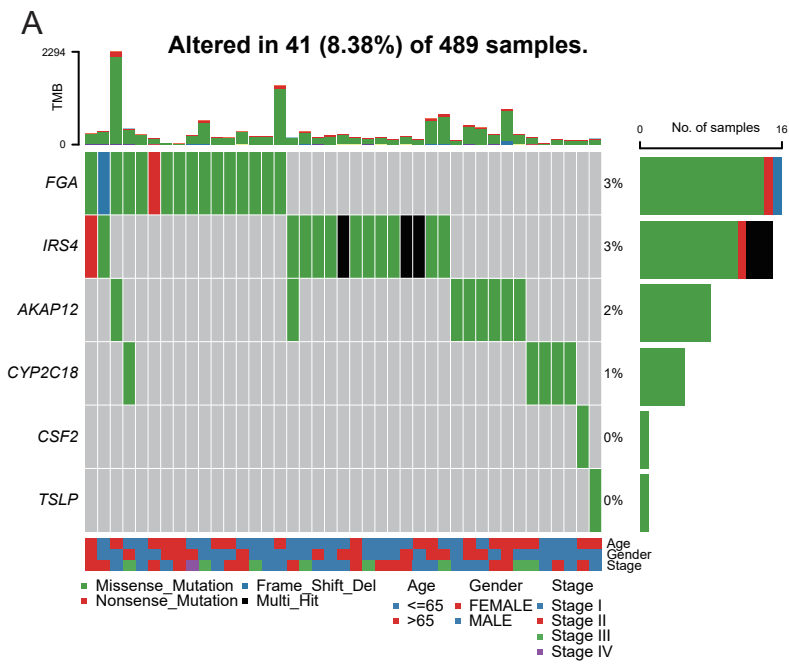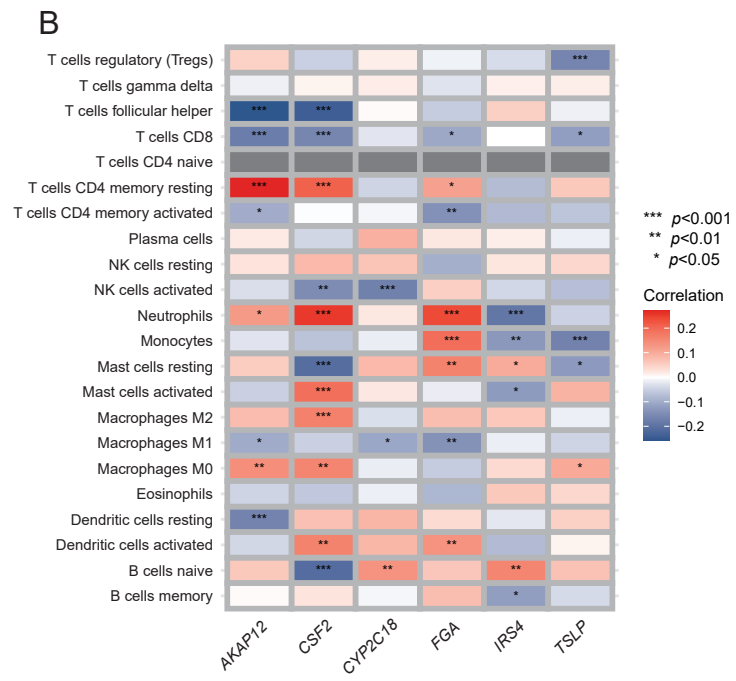

Supplement: Supplementary file 3 [file DataSheet1.zip › Supplementary Figures/S8.pdf]

Checkpoint

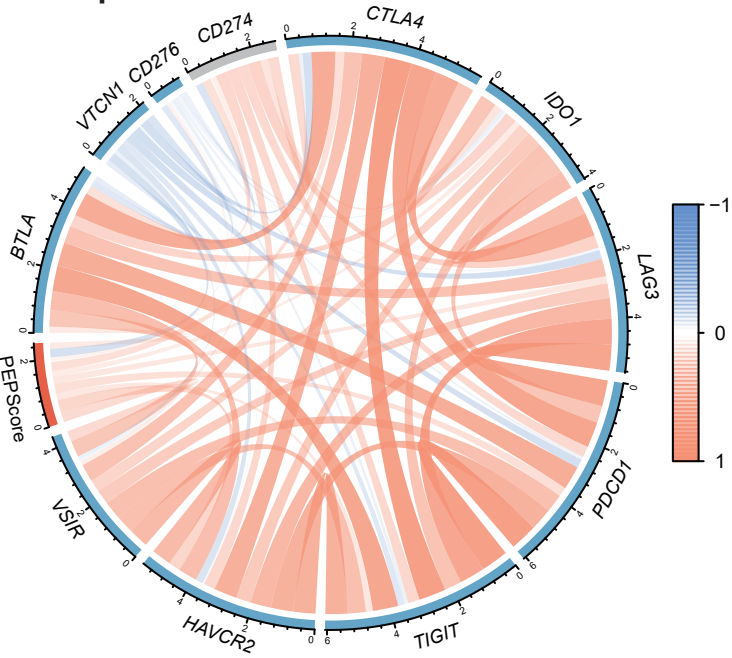

Chemokine

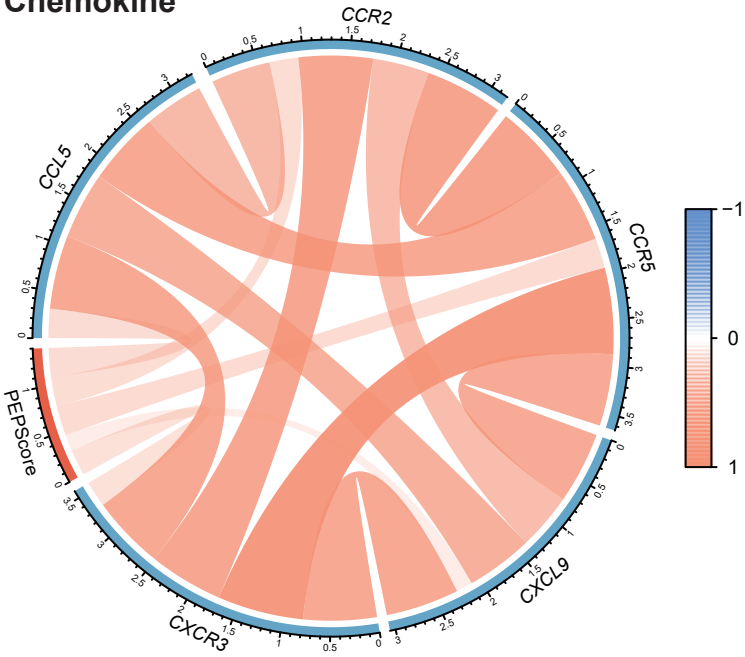

Supplement: Supplementary file 3 [file DataSheet1.zip › Supplementary Figures/S9.pdf]
