# Supplementary material for: A pyroptosis expression pattern score predicts prognosis and immune microenvironment of lung squamous cell carcinoma
Source: Front Genet. 2022 Nov 10;13:996444. doi: 10.3389/fgene.2022.996444 (PMC9685532; doi:10.3389/fgene.2022.996444)
Supplement: Supplementary file 3 [file S3.pdf › Supplementary Figures/S3.pdf]

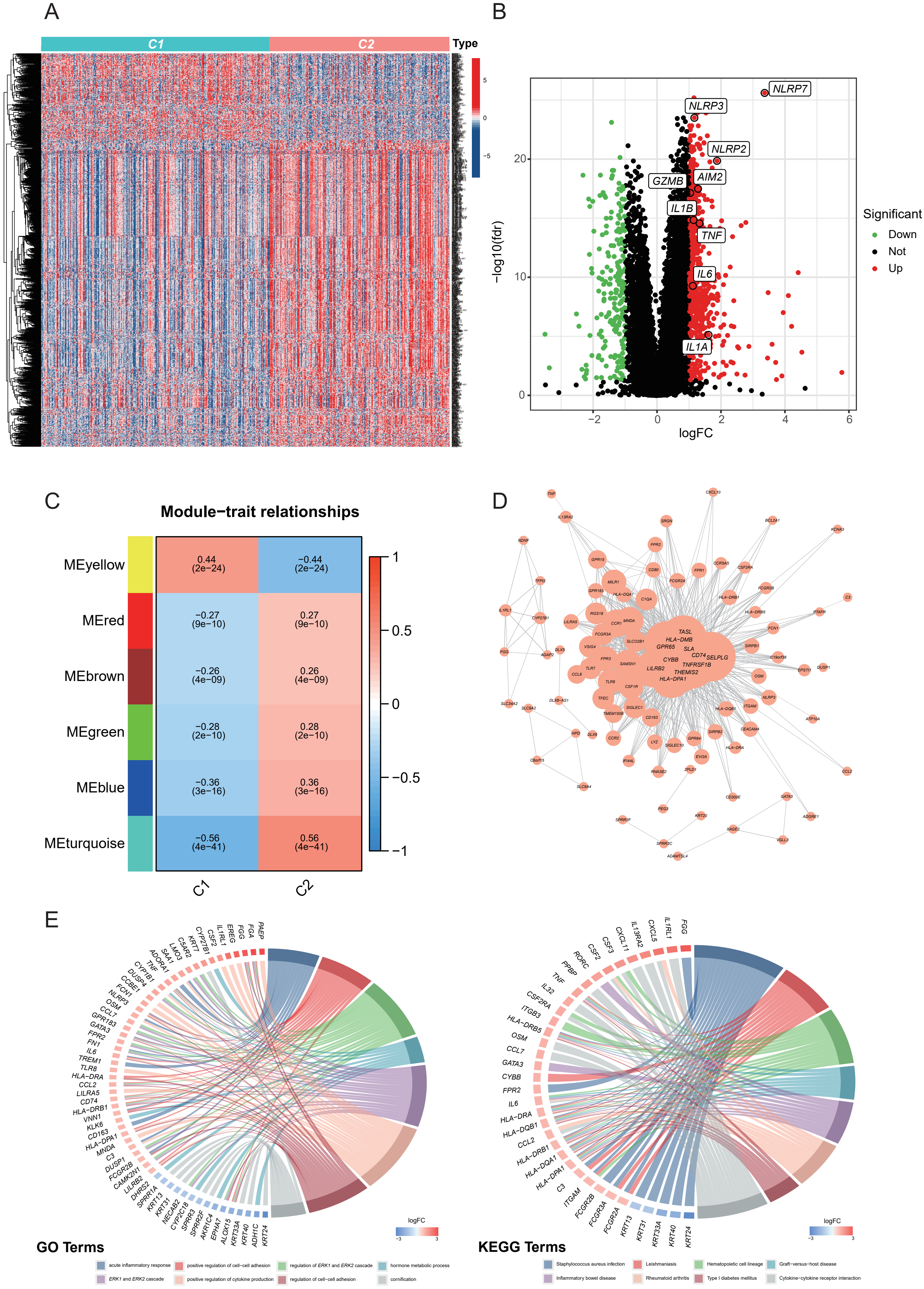
A
C1
C2
Type
5
0
−5
B
NLRP7
NLRP3
NLRP2
GZMB
AIM2
IL1B
TNF
IL6
IL1A
−log10(fdr)
logFC
Significant
Down
Not
Up
C
Module−trait relationships
MEyellow
MEred
MEbrown
MEgreen
MEblue
MEturquoise
0.44 (2e−24)
−0.44 (2e−24)
−0.27 (9e−10)
0.27 (9e−10)
−0.26 (4e−09)
0.26 (4e−09)
−0.28 (2e−10)
0.28 (2e−10)
−0.36 (3e−16)
0.36 (3e−16)
−0.56 (4e−41)
0.56 (4e−41)
C1
C2
D
TASL
HLA−DMB
GPR65
SLA
CD74
SELPLG
CYBB
TNFRSF1B
LILRB2
THEMIS2
HLA−DPA1
E
GO Terms
acute inflammatory response
positive regulation of cell−cell adhesion
regulation of ERK1 and ERK2 cascade
hormone metabolic process
ERK1 and ERK2 cascade
positive regulation of cytokine production
regulation of cell−cell adhesion
cornification
KEGG Terms
Staphylococcus aureus infection
Leishmaniasis
Hematopoietic cell lineage
Graft−versus−host disease
Inflammatory bowel disease
Rheumatoid arthritis
Type I diabetes mellitus
Cytokine−cytokine receptor interaction
logFC
